# An Improved Approach for RSSI-Based only Calibration-Free Real-Time Indoor Localization on IEEE 802.11 and 802.15.4 Wireless Networks

**DOI:** 10.3390/s17040717

**Published:** 2017-03-29

**Authors:** Marco Passafiume, Stefano Maddio, Alessandro Cidronali

**Affiliations:** Department of Information Engineering, University of Florence, Florence 50139, Italy; stefano.maddio@unifi.it (S.M.); alessandro.cidronali@unifi.it (A.C.)

**Keywords:** indoor localization, RSSI, WSN, WiFi, 802.11, 802.15.4, bluetooth, iBeacon, iLocate, phaseless, COTS, network, DoA, fingerprinting, tracking

## Abstract

Assuming a reliable and responsive spatial contextualization service is a must-have in IEEE 802.11 and 802.15.4 wireless networks, a suitable approach consists of the implementation of localization capabilities, as an additional application layer to the communication protocol stack. Considering the applicative scenario where satellite-based positioning applications are denied, such as indoor environments, and excluding data packet arrivals time measurements due to lack of time resolution, received signal strength indicator (RSSI) measurements, obtained according to IEEE 802.11 and 802.15.4 data access technologies, are the unique data sources suitable for indoor geo-referencing using COTS devices. In the existing literature, many RSSI based localization systems are introduced and experimentally validated, nevertheless they require periodic calibrations and significant information fusion from different sensors that dramatically decrease overall systems reliability and their effective availability. This motivates the work presented in this paper, which introduces an approach for an RSSI-based calibration-free and real-time indoor localization. While switched-beam array-based hardware (compliant with IEEE 802.15.4 router functionality) has already been presented by the author, the focus of this paper is the creation of an algorithmic layer for use with the pre-existing hardware capable to enable full localization and data contextualization over a standard 802.15.4 wireless sensor network using only RSSI information without the need of lengthy offline calibration phase. System validation reports the localization results in a typical indoor site, where the system has shown high accuracy, leading to a sub-metrical overall mean error and an almost 100% site coverage within 1 m localization error.

## 1. Introduction

Indoor localization is one of the most challenging goals for mobile device application development, as evidenced by the growing interest resulting in the birth of different consortia (i.e., i-Locate [1]) and coarse wireless devices (i.e., Apple iBeacon [2], NexTOme [3]) with simple software development kits (SDKs). In [4], some achievements resulting from the worldwide Microsoft Indoor Localization Competition are outlined. Note that all the proposed systems were developed with certain constraints, including cost-effectiveness, configuration speed and transparency.

Particular attention has been given to infrastructure-free systems [4]; that are systems used by most widespread Component-Off-The-Shelf (COTS) devices (i.e., smartphones, tablets, etc.), which only implement standard communication protocols and achieve localization services, starting from coarse and protocol estimated parameters (i.e., RSSI, LQI). In fact, localization systems compatible with typical user devices are considered the only answer to the development of friendly, cost-effective and simple localization [5]. Therefore, improving localization accuracy is achievable by refining localization algorithms.

In terms of IEEE 802.11 and 802.15.4 compliant systems, the direct physical parameters for packet transmission are the time of arrival (ToA) and the received signal strength indicator (RSSI). Time difference of arrival (TDoA) techniques can produce interesting results [6], and they may be the solution; however, as shown in [6,7] the lack of sufficient timing for resolutions at the protocola data level impose the Component-Off-The-Shelf (COTS) transceiver architecture to open up and achieve more accurate time estimations (likely using higher frequency ADCs) at a lower protocol stack layer. If such modifications could be considered, hardware adaptation would require higher frequency ADCs and DACs, comporting all related mixed-signal hardware changes and incrementing costs. With respect to the constraints of implementing COTS transceivers, the system design can take advantage of the RSSI parameter estimation available in all the IEEE 802.11 and 802.15.4 implementations. The direct RSSI evaluation appears to be an unreliable measure [8] for achieving a sufficient accurate localization in indoor environments. By this, some RSSI-based solutions based on distributed network of routers have been proposed in the past, applying fingerprint-like methods [4,9,10] or trilateration by range estimation [11,12]: in all of these solutions coarse errors arise due to the unpredictability of RSSI estimations in complex environments. Localization accuracy is typically improved by obtaining additional information from user devices’ inertial sensors and by applying Kalman filtering [13,14,15], but information fusion requires high computational power and accuracy, both directly influencing the overall localization performance. The best localization accuracy is achieved through fingerprint methods, but a complex off-line calibration phase shall be introduced to make the system operational: such calibration strictly depends on particular environment characteristics (i.e., routers distribution, furnishings distribution, etc.), so it makes the overall localization solution very complex to be installed and managed.

This work aims to propose an IEEE 802.11/802.15.4 network compliant indoor localization system, which is capable of achieving sub-metrical accuracy without any kind of off-line calibration phases. The proposed approach is based on a network of anchor nodes (or rather, typical routers) based on a particular SBA (Switched Beam Antenna) structure [16,17] which is capable of SDMA (Space Division Multiplexing Access).

In Section 2 we demonstrate that such anchor node is able to provide a more predictable radiation pattern distribution across the area, and through SDMA, exploiting more co-operative anchor nodes, the resulting constellation is able to subdivide overall area in small cells thus enabling a coarse metrical space subdivision. In this refined space domain, the proposed localization algorithm estimates effectively the target position. In force of the pattern predictability and space cell subdivision, through a RSSI-based fingerprint-like localization algorithm based on a purely ideal “reference map”, the proposed system is able to achieve the sub-metrical localization accuracy for both static and mobile target nodes. Because proposed localization algorithm is based only on RSSI estimations, no more than a typical 802.11/802.15.4 transceiver is required while RSSI values are obtained in a fully transparent way during standard packet network communication.

## 2. Proposed Hardware Infrastructure

**Figure 1:**
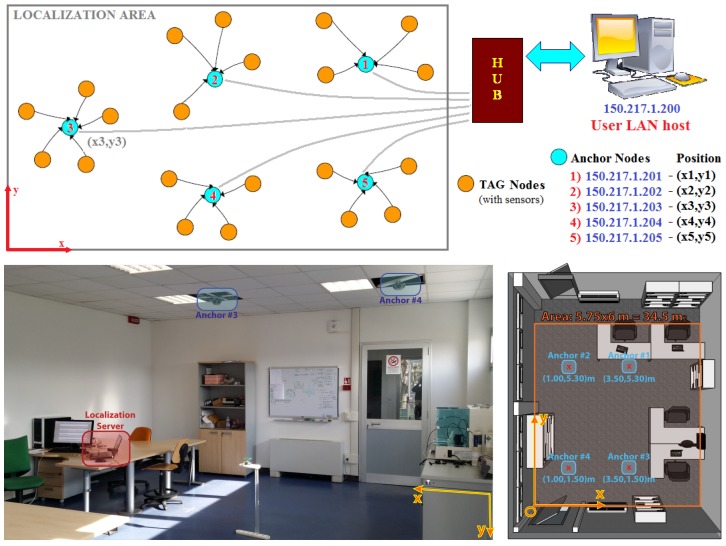
Distributed 802.15.4 Anchors network infrastructure and experiment site.

In [17,18], COTS-only hardware for transparent indoor localization was proposed for use in a distributed network of IEEE 802.15.4 anchor nodes hanging from the ceiling, which offer Ethernet-to-ZigBee connectivity. Every anchor node is capable of transferring packets between any LAN host and each ZigBee node (Figure 1), while the LAN host collects all the localization-related data.

A reference table for complete anchor networks is built in the LAN host, containing the position of each anchor node. As shown in [18,19], an ideal two-dimensional map of the expected RSSI is collected for each antenna and anchor (considering the user node as the transmitter), directly projecting each oriented polar ideal antenna pattern to the plane, with the height being the mean height of the TAG nodes (i.e., a typical height of 1.10 m from the floor is considered).

**Figure 2:**
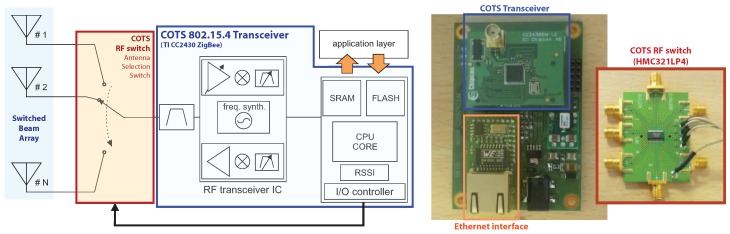
COTS IEEE 802.15.4 anchor node hardware description/motherboard.

Each anchor node is built on the concept of a switched-beam array [16,20,21]. In Figure 2, a brief hardware description is shown, including both the antenna array structure and the anchor node block diagram; functionally, the concept is to place a uC-controlled RF switch on a standard ZigBee transceiver (i.e., in [19], a COTS Texas Instruments CC2430 transceiver was used), which connects the RF channel to every antenna of the array.

**Figure 3:**
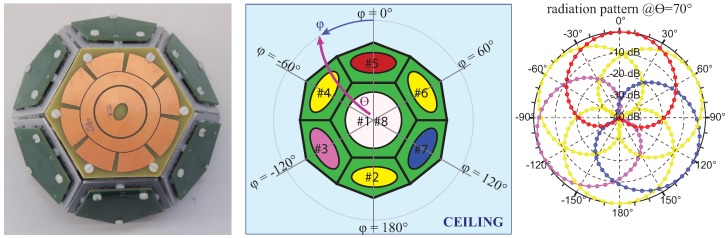
Switched-beam array anchor node [17].

The antennas are implemented as printed patch antennas, radiating a characteristic and regular far-field pattern (Figure 3, [22,23,24]). The antennas operate in circular polarization, permitting a reliable link regardless of the relative orientation of the tag [19,25,26]. Furthermore, circular polarization is a strong aid in contrasting multipath impairment [27,28,29]. A set of patterns is projected over different spatial areas [19] and for each anchor tag data packet a complete RSSI vector for each antenna, called the steering vector, is given.

### 2.1. Proposed Localization Method

A steering vector defined through RSSI values contains information only about packet received signal power, thus phase information is totally unavailable. Despite this an accurate design of the array structure (Figure 2, [16,30]) can provide an excellent pattern differentiation throughout each single anchor domain, thus lowering the expected Cramer-Rao Bound lower limit for localization accuracy [31] and enabling the single anchor node to perform Direction-of-Arrival estimation. In [32] a specific implementation of the DoA MuSiC algorithm [33] has been proposed for phase-less RSSI steering vectors. Such an algorithm was successfully implemented in the 802.15.4 COTS based SBA designed in [16] and proposed in Figure 3, showing that a completely phaseless and RSSI-only based architecture can perform DoA localization [17].

Different anchors DoA estimations could be used to perform a three dimensional localization overall a site, but RSSI DoA estimation is far from being accurate enough to achieve a limited dilution of precision for localization in large areas. To improve overall accuracy some enhanced triangulation algorithms exist [34], but the actual problem is that each different DoA estimation is affected by an angle estimation error while such estimations are applied as arguments of strictly non-linear trigonometrical function to perform final $\left(x,y\right)$ estimation [35].

Dealing with a network of N distributed anchors, making the final localization using N different and independent DoA estimation without considering any kind of relationship between them does not exploit the entire information available. A stronger control over estimation error propagation can be achieved by applying an estimation algorithm over the entire set of RSSI data given by the entire set of installed anchors: the effective increase of information quantity available to the final localization algorithm allows to complete reciprocal anchor observations thus reducing overall estimation error.

**Figure 4:**
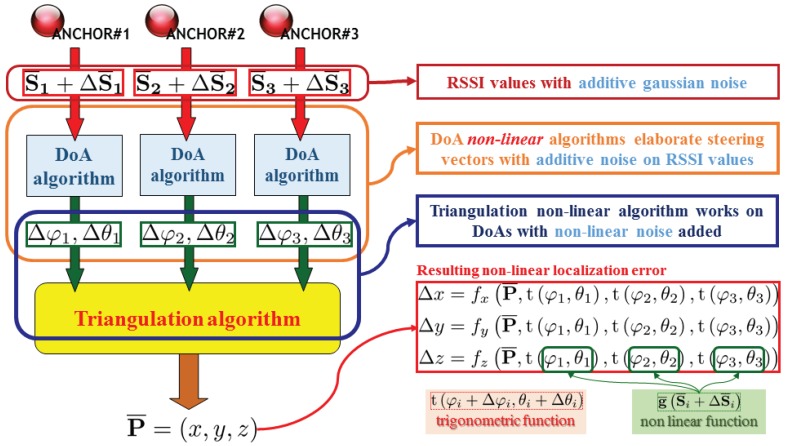
Error propagation scheme for RSSI Direction-of-Arrival (DoA) based localization.

**Figure 5:**
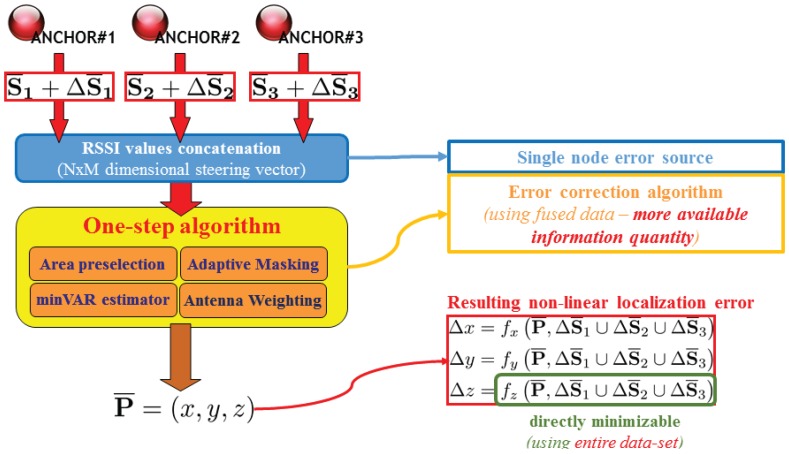
Error propagation scheme for RSSI DoA-fused data localization.

The error propagation scheme is conceptually depicted in Figure 4 and Figure 5. Note that while the typical triangulation approach in fact gets the different DoA estimations from each anchor node without performing any kind of reciprocity check, dealing with overall RSSI information (thus processing a “global steering vector” given by the entire network of anchors) a single conceptual block can access to the entire information batch allowing to implement smarter localization algorithms.

Note that the error propagation model of Figure 4 is still valid for trilateration approaches [36], as the Friis formula inversion is required to estimate the distance between the anchor and each node. In this case, the RSSI measurements error can have an even bigger impact.

Figure 4 reveals how the main source of weakness is caused by a pair of non-linear transformations applied over the collected steering vector, which is affected by RSSI measurement errors modelled as a Gaussian noise distribution [32]. Note that the non-linear function of the localization error depends on non-linear functions $\bar{g}\left(\Delta {\bar{S}}_{i}\right)$ applied as trigonometric function $t\left(\varphi ,\theta \right)$ arguments.

To achieve an higher control over error propagation, a one-step localization algorithm is highly preferable. In Figure 5, note that the localization error function becomes directly dependent on RSSI measurement errors, thus final localization error can be better controlled by refining the direct localization estimator function. In one-step localization, error propagation does not depend on trigonometrical functions, and the overall information comes from a distribute set of directive antennas (grouped by anchor nodes), building a more descriptive and fully exploitable data set.

The proposed approach analyses the RSSI values collected from the overall anchor-node antennas. Dealing with an highly spread antenna distribution, an extensive information about environment is given reducing the needing of information fusion with additional sensor data; the needing of an off-line calibration phase is removed thanks to predictability increase overall the observation area. Additional information given by a “global steering vector” can be exploited through different processing blocks (accurately described in Section 3) lying within the “One-Step” localization algorithm (Figure 5) which are able to feed each other to refine final estimation results.

In summary, the hardware architecture shown in [17,19] was used, the algorithmic layer is deeply different from a simple implementation of the DoA RSSI algorithm on every anchor, obtaining the final spatial localization through the simple triangulation algorithm, as shown in [35]. In [19], each M-dimensional steering vector collected for each of the N = 4 anchor nodes (each composed by M = 7 antenna elements) was used to compose a single MxN-dimensional global steering vector, which became the input of the direct localization algorithm placed on the server (Figure 1).

## 3. One-Step Localization Algorithm

In Section 2.1 the localization method has been introduced. As it is depicted in Figure 5 the core of actual localization approach relies within the “One-Step algorithm” block: This paragraph will describe block implementation in depth, thus evaluating algorithm improvements respect RSSI measure noise.

In the “one-step” approach the effective steering vector is the vector containing every RSSI collected from each antenna of every anchor node, and it becomes a global steering vector. Global steering vectors correspond to a long concatenation of all the different steering vectors collected from each anchor node placed in a known order (e.g., lexicographical order based on the name associated with each anchor). For each packet transfer, the host gets a global steering vector, as shown in Equation (1).

$$\bar{S}=\left(\begin{array}{c} {\bar{S}}_{1}\\ {\bar{S}}_{2}\\ \vdots \\ {\bar{S}}_{N} \end{array}\right)⇐\left\{\begin{array}{c} \text{“anchor\#1”}\\ \text{“anchor\#2”}\\ \vdots \\ \text{“anchor\#N”} \end{array}\right.\;\mathrm{with}\;{\bar{S}}_{i}=\left(\begin{array}{c} s_{1i}\\ s_{2i}\\ \vdots \\ s_{M_{i}i} \end{array}\right)\;i-\mathrm{th}\;\mathrm{anchor}\;\mathrm{steering}\;\mathrm{vector}\;(M\;\mathrm{antennas})$$

(1) $$\mathrm{or}\;\;\bar{S}={\langle \underset{\mathrm{number}\;\mathrm{of}\;\mathrm{elems}=\sum_{i=1}^{N}M_{i}=M\times N}{\underset{︸}{s_{11}|s_{21}\left|\ldots \right|s_{M_{1}1}|s_{12}|s_{22}\left|\ldots \right|s_{M_{2}2}\left|\ldots \right|s_{1N}\left|\ldots \right|s_{M_{N}N}}}\rangle }^{H}$$

Next to the steering vector is a reference map of the whole set of expected global steering vectors for each different position in the localization space (Equation (2)).

(2) $${\bar{S}}_{collected}=\underset{\begin{array}{c} \mathrm{expected}\;\mathrm{ideal}\\ \mathrm{antenna}\;\mathrm{gains} \end{array}}{\underset{⎵}{M\left(x,y\right)}}+\underset{\begin{array}{c} \mathrm{real}\;\mathrm{vs}.\;\mathrm{ideal}\\ \mathrm{projection}\;\mathrm{bias} \end{array}}{\underset{⎵}{\overset{M_{\Delta }\left(x,y\right)}{\overset{⎴}{M_{\mathrm{BIAS}}\left(x,y\right)+P_{\mathrm{TX}}}}}}\;\forall \left(x,y\right)\mathrm{in}\;\mathrm{the}\;\mathrm{localization}\;\mathrm{domain}$$

A generic Maximum-Likelihood fingerprinting algorithm is based on finding the solution to the problem in Equation (3) [19,31,37]. The $C\left(x,y\right)$ function is called the pseudospectrum function or the estimator and is defined as a $R^{2}\to R$ function in the localization domain.

(3) $$\left(\hat{x},\hat{y}\right)={\mathrm{argmin}}_{\left(x,y\right)}\left.C\left(x,y\right)\right.\;\mathrm{with}C\left(x,y\right)=F\left(\bar{S},M\left(x,y\right)\right)$$

Maximum likelihood (ML) algorithms differ depending on the estimator. Among the ML estimators, a reduced computational cost subclass can be defined; the generic form is shown in Equation (4).

(4) $$\left\{\begin{array}{c} C\left(x,y\right)=F\left(\bar{S},M\left(x,y\right)\right)\\ C\left(\hat{x},\hat{y}\right)=\min\left[C\left(x,y\right)\right] \end{array}\right.\;\Rightarrow \;\left\{\begin{array}{c} C\left(x,y\right)=F\left(\bar{S}-M\left(x,y\right)\right)\geq 0\\ C\left(\hat{x},\hat{y}\right)=F\left(\bar{S}-M\left(\hat{x},\hat{y}\right)\right)=0\\ F\left(\bar{x}\right)=\sum_{i=1}^{N_{x}}F_{i}\left(\bar{x}\right) \end{array}\right.$$

This paper will cover only reduced computational cost estimators to follow the imposed real-time constraint. In Section 5, the localization results will be compared between the proposed estimators and the State-of-Art, computationally complex MUSIC estimator [32,33]. The least squares estimator is the simplest ML estimator and is the one referred to in [19].

(5) $$C\left(x,y\right)={\left|\left|\bar{S}-M\left(x,y\right)\right|\right|}^{2}=\sum_{i=1}^{M\times N}{\left(s_{i}-m_{i}\right)}^{2}$$

Following a classic fingerprinting approach [10,38], in [19], the concept of predicted fingerprinting is introduced to achieve an acceptable localization accuracy in the site shown in Figure 1. In the predicted fingerprinting reference map, which is compounded by steering vectors collected at each position in classic fingerprinting approaches, the software projects every antenna pattern of each anchor onto the observation floor space. This step was the novelty of [19], as, at the time, there was no need for an extensive offline calibration phase prior to a system’s effective utilization by replacing it with an a posteriori tuning/optimization of antenna projection map parameters [38] respect a small set of training observations.

Note that, as shown in [19], antenna projections are built considering a reference height of 1.10 m, the typical height for mobile phone use when the user is standing.

### 3.1. Area Preselection

**Figure 6:**
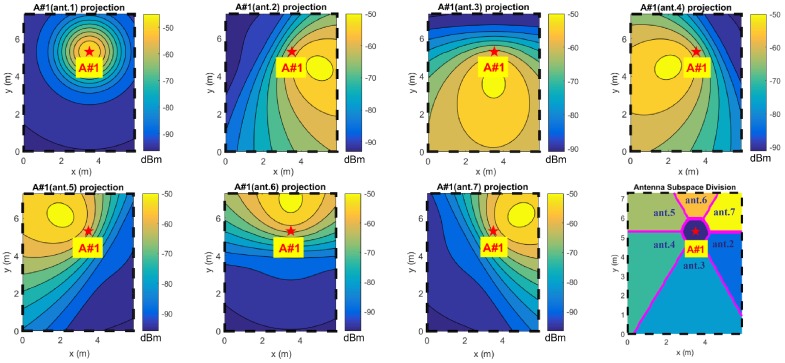
Predicted fingerprinting map for the first anchor node.

The reliability of the estimator values is directly related to the reliability of reference map $M\left(x,y\right)$ and the function trends can be dramatically altered by RSSI measurement noise; arguments bias can effectively produce some wrong relative minimums, which can become new absolute minimums that alter the final localization estimation. One way of keeping this source of estimation bias under control is exploiting the capability of each anchor to make a coarse spatial subdivision for the area of competence [19,39] (Figure 6).

**Figure 7:**
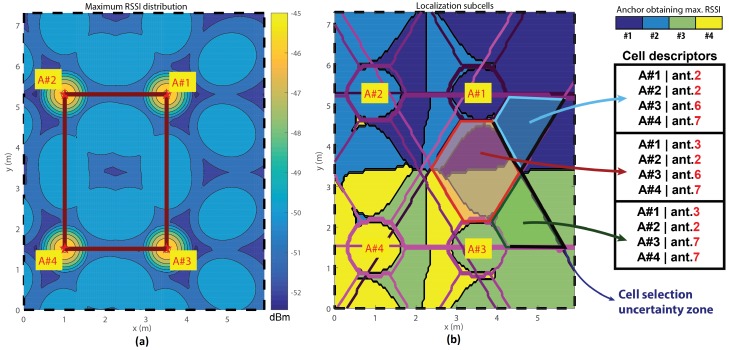
Maximum RSSI distribution over the site (**a**) and related cell descriptors (**b**).

In the subdivided localization spaces of multiple anchors in smaller sub-zones (or cells), each anchor is uniquely linked to a list of corresponding maximal antennas for each anchor plus the absolute maximal antenna (Figure 7). Therefore, every steering vector directly links to a subselection of localization domain (or rather a sub-cell), reducing the computational cost and the maximal localization error.

Defining a preselection steering vector with related preselection steering vectors reference map as in Equation (6), the preselection algorithm is shown in Equation (7).

(6) $$\bar{P}={\langle {\mathrm{id}}_{1}|{\mathrm{id}}_{2}\left|\ldots \right|{\mathrm{id}}_{N}\rangle }^{H}\;\mathrm{with}{\mathrm{id}}_{i}=\max.\;\mathrm{antenna}\;\mathrm{id}\;\mathrm{on}\;i-\mathrm{th}\;\mathrm{anchor}$$

$$P_{\mathrm{map}}={\bar{P}}_{expected}\;\forall \left(x,y\right)\;\mathrm{in}\;\mathrm{the}\;\mathrm{localization}\;\mathrm{domain}$$

The preselection algorithm removes each point from the reference map which does not belong in the subselection condition represented by the masking function $W\left(x,y\right)$: $R^{2}\to \left\{0,1\right\}$.

The subdomain reference map equals

(7) $$M_{\mathrm{sel}}\left(x,y\right)=M\left(x,y\right)\cdot W\left(x,y\right)\;\mathrm{with}\;W\left(x,y\right)=\left\{\begin{array}{c} 1\;\mathrm{when}\;\overset{\mathrm{cell}\;\mathrm{ID}\;\mathrm{distance}\;\mathrm{function}}{\overset{⎴}{d\left(\bar{P},P_{\mathrm{map}}\left(x,y\right)\right)}}\leq d_{\max}\\ 0\;\mathrm{otherwise} \end{array}\right.$$

with the cell identifier distance function $d\left(\bar{P},P_{\mathrm{map}}\left(x,y\right)\right)$ defined in Equation (8).

(8) $$d:N^{N}\to N^{}\;/\;d\left(\bar{v},\bar{w}\right)={\left|v_{i}-w_{i}\right|}_{\max}$$

The preselection algorithm relies on unrelated sub-domain removal (i.e., unrelated to selected cells), which shows cell identifiers too different or “far” from the one extracted from the measured data.

Rough area preselection can lead to localization mistakes due to the preselection uncertainty for areas laying near cell boundaries. The feature is associated with an index called the maximum antenna distance ($d_{\max}$), which permits the choice of the strictness of cell selection. For an example, looking at Figure 7b, if the node position belongs near the red-coloured border, a cell selection mistake could lead to increased localization errors. An effective workaround is to incorporate the cells that belong on adjacent antennas in the domain selection. Imposing a higher $d_{\max}$ causes a reduction in domain selection selectivity, so a complete localization routine could allow for an adaptive algorithm to increment the $d_{\max}$ only when needed.

### 3.2. Adaptive Masking

If the localization problem is stated as in Equation (3), the main condition for the estimator function is to be convex inside the current localization domain. By this, the presence of a possible correct estimation is identified only if an absolute minimum is present in the observed domain.

The absolute minimum is defined for a $R^{2}\to R$ function as the point $\left(\hat{x},\hat{y}\right)$ corresponding to the minimum value, in which the gradient and the Hessian matrix are defined in the neighborhood and the conditions in Equation (9) is verified.

(9) $$\left\{\begin{array}{c} \;\;C\left(\hat{x},\hat{y}\right)\in C^{2}\\ \;\;\;\;↓\\ \exists \varepsilon >0:\forall \left(x,y\right)\;/\;\\ \;\left|\left|\left(x,y\right)-\left(\hat{x},\hat{y}\right)\right|\right|<\varepsilon \\ \underset{C^{2}\;\mathrm{necessary}\;\mathrm{condition}}{\underset{⎵}{\;\;C\left(x,y\right)\;\mathrm{defined}\;\;}} \end{array}\right.\Rightarrow \left\{\begin{array}{c} {\left[\nabla_{}C\left(x,y\right)\right]}_{\left(\hat{x},\hat{y}\right)}=\left(\begin{array}{c} {\left[\frac{\partial C\left(x,y\right)}{\partial x}\right]}_{\left(\hat{x},\hat{y}\right)}\\ {\left[\frac{\partial C\left(x,y\right)}{\partial y}\right]}_{\left(\hat{x},\hat{y}\right)} \end{array}\right)=\bar{0}\\ {\left[\frac{\partial^{2}C\left(x,y\right)}{\partial x^{2}}\right]}_{\left(\hat{x},\hat{y}\right)}>0\\ {\left[\Delta H\left(x,y\right)\right]}_{\left(\hat{x},\hat{y}\right)}={\left|\begin{array}{cc} \frac{\partial^{2}C\left(x,y\right)}{\partial x^{2}} & \frac{\partial^{2}C\left(x,y\right)}{\partial x\partial y}\\ \frac{\partial^{2}C\left(x,y\right)}{\partial y\partial x} & \frac{\partial^{2}C\left(x,y\right)}{\partial y^{2}} \end{array}\right|}_{\left(\hat{x},\hat{y}\right)}>0 \end{array}\right.$$

**Figure 8:**
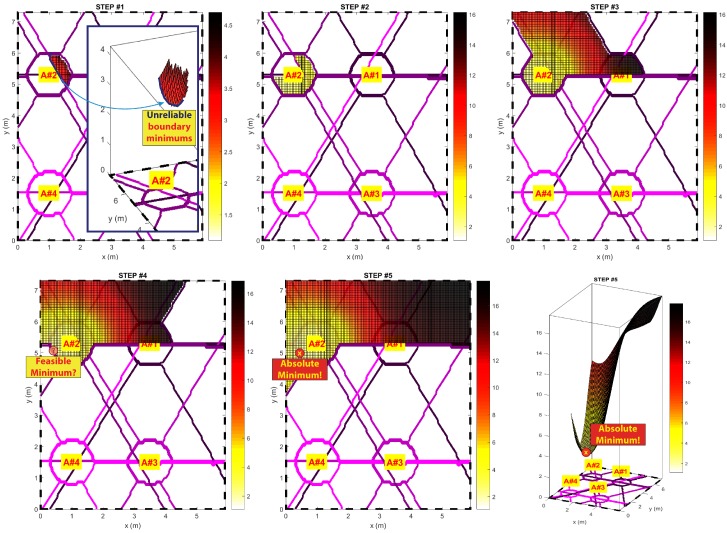
Example of pseudospectrum $C\left(x,y\right)$ composition using an adaptive masking algorithm.

The approximate solution of the statement in Equation (3) is given computationally by looking for the indeces matching the minimum value of the numerically computed pseudospectrum function; therefore, the direct check of the conditions in Equation (9) is unnecessary. Nevertheless, if the selected subdomain ends before the pseudospectrum value reaches its absolute minimum, the preselection feature can lead to serious estimation errors (Figure 8).

Making the subdomain selection algorithm adaptive and dependent on each observation could be a valid workaround. Since an a priori knowledge about the maximum position does not exist, an one-step definition of the subdomain mask is unreliable. The idea is to apply an iterative algorithm that verifies whether the minimum relies on an area that is safe and far enough from any subdomain edge for each execution. If not, the subdomain boundary will be extended to enlarge the selection area, increasing the $d_{\max}$ parameter shown in Equation (7) and reapplying the mask. An example pseudocode implementation is shown below. Figure 8 shows the progressive building of the pseudospectrum using the adaptive masking algorithm.

In Algorithm 1, the argmin operator is numerically computed, so Equation (9) computation is unnecessary. Thus, the “CheckReliabilityMin” function must only check that the computed minimum does not belong to the masked domain edges. If the condition is true, Equation (9) will be implicitly verified thanks to the pseudospectrum continuity of the domain (Equation (5)) [19].

**Algorithm 1** Localization Algorithm with Adaptive Masking.1:**function** Locate–2D2:    $\left(\mathrm{reliableMin}\leftarrow 0\right)\cup \left(d_{\max}\leftarrow 0\right)$3:    $\bar{S}\leftarrow$ GetFusedSVector()                   ▹ get data from the network (Equation (1))4:    $\bar{P}\leftarrow$ GenerateCellIdentifier($\bar{S}$)            ▹ generate the preselection vector $\bar{P}$ (Equation (6))5:    M← GetReferenceMap()           ▹ get the steering vector reference map M (Equation (2))6:    $P_{\mathrm{map}}\leftarrow$ GetCellsMap()       ▹ get the preselection vector reference map $P_{\mathrm{map}}$ (Equation (6))7:    8:    **while** $\mathrm{not}\left(\mathrm{reliableMin}\right)$ **do**9:        $M_{\mathrm{sel}}\leftarrow$ GetCellDomain($M,P_{\mathrm{map}},\bar{P},d_{\max}$)   ▹ apply the map masking M·W (Equation (7))10:        C ← Calculatepseudospectrum($\bar{S},M_{\mathrm{sel}}$)       ▹ compute the $C\left[i,j\right]$ function (Equation (5))11:        $\left[\hat{i},\hat{j}\right]\leftarrow {\mathrm{argmin}}_{\left[i,j\right]}\left.C\left[i,j\right]\right.$       ▹$\left[\hat{i},\hat{j}\right]$ = indeces of the $C\left[i,j\right]$ minimum (Equation (3))12:        reliableMin←CheckReliabilityMin(C,$\left[\hat{i},\hat{j}\right]$)      ▹ verify Equation (9) domain conditions(Algorithm 2)13:        **if** $\mathrm{not}\left(\mathrm{reliableMin}\right)$ **then**14:           $d_{\max}\leftarrow d_{\max}+1$         ▹ increase the max. acceptable cell ID distance (Equation (8))15:        **end** **if**16:    **end** **while**17:    18:    $\left(\hat{x},\hat{y}\right)\leftarrow \left(M_{\mathrm{sel}}.\mathrm{xVector}\left[\hat{i}\right],M_{\mathrm{sel}}.\mathrm{yVector}\left[\hat{j}\right]\right)$  ▹ extract the $\left(\hat{x},\hat{y}\right)$ from the indexed domain19:    **return** $\left(\hat{x},\hat{y}\right)$20:**end function**

To minimize the computational cost, it is best to ignore all the pseudospectrum points that belong outside of the masked domain. This marks all the points where the masking function is null (W$\left[i,j\right]$ = 0 - Equation (7)) as NaNs (i.e., MATLAB’s Not-a-Number marker). Marking the value of a matrix as a NaN makes it a non-existent value, so any further processing propagates the non-existence condition.

An implementation for the “CheckReliabilityMin” function is shown in Algorithm 2. The simplest way to check a condition in a point neighborhood is to write a nested cycle where, for each column of the matrix, all the rows are checked, so the neighbor zone will be square shaped.

**Algorithm 2** CheckReliabilityMin Function.1:**function** CheckReliabilityMin(C,$\left[\hat{i},\hat{j}\right]$)2:    let radius= $k_{0}\cdot \min\left[\mathrm{length}\left(M.\mathrm{xVector}\right),\mathrm{length}\left(M.\mathrm{yVector}\right)\right]$3:    4:    **for** $i=\left(\hat{i}-radius\right)$ to $\left(\hat{i}+radius\right)$ **do**5:        **for** $j=\left(\hat{j}-radius\right)$ to $\left(\hat{j}+radius\right)$ **do**6:           **if** C$\left[i,j\right]$=NaN **then**            ▹ point near the domain boundary7:               **return** 0 ▹ pseudospectrum is not defined overall in the $\left(\hat{x},\hat{y}\right)$ neighborhood8:           **end** **if**             ▹ high risk of minimum identification mistakes9:        **end** **for**10:    **end** **for**11:    12:    **return** 1              ▹ point far enough from the domain boundary13:**end** **function**

A square domain neighbour does not respect the mathematical definition of the neighbourhood of a point (defined as a circular area), but a workaround is to check the condition over an area that contains a typical neighborhood.

The radius of the point’s neighbour is defined through a proportionality constant number of points over the localization domain’s minor dimension (conventionally $k_{0}=0.1$); the process for checking for an effective domain is shown below.

$$\left\{\begin{array}{c} \Delta x=\frac{\max M.\mathrm{xVector}-\min M.\mathrm{xVector}}{\mathrm{length}\left(M.\mathrm{xVector}\right)}\;\Delta y=\frac{\max M.\mathrm{yVector}-\min M.\mathrm{yVector}}{\mathrm{length}\left(M.\mathrm{yVector}\right)}\;\Delta =\underset{\left(x,y\right)\;\mathrm{sample}\;\mathrm{steps}}{\underset{⎵}{\min\left[\left[\Delta x,\Delta y\right]\right]}}\\ D=\left\{\left(x,y\right)\in R^{2}:\left|\left|\left(x,y\right)-\left(\hat{x},\hat{y}\right)\right|\right|<\left(\mathrm{radius}\cdot \Delta \right)\right\}\;\mathrm{standard}\;R^{2}\;\mathrm{neighbor}\;\mathrm{definition} \end{array}\right.$$

(10) $$\left.\begin{array}{c} \underset{⇓}{\underset{⎵}{\overset{⇓}{\overset{⎴}{D_{k}=\left\{\left[i,j\right]\in N^{2}:\left(\left|\left|i-\hat{i}\right|\right|<\mathrm{radius}\right)\cap \left(\left|\left|j-\hat{j}\right|\right|<\mathrm{radius}\right)\right\}}}}}\\ \left\{\begin{array}{c} x=M.\mathrm{xVector}\left[i\right],\;\hat{x}=M.\mathrm{xVector}\left[\hat{i}\right]\\ y=M.\mathrm{yVector}\left[j\right],\;\hat{y}=M.\mathrm{yVector}\left[\hat{j}\right]\\ \varepsilon_{x}=\mathrm{radius}\cdot \Delta x,\;\varepsilon_{y}=\mathrm{radius}\cdot \Delta y \end{array}\right.\\ \overset{⇓}{\overset{⎴}{D_{o}=\left\{\left(x,y\right)\in R^{2}:\left(\left|\left|x-\hat{x}\right|\right|<\varepsilon_{x}\right)\cap \left(\left|\left|y-\hat{y}\right|\right|<\varepsilon_{y}\right)\right\}}} \end{array}\right.$$

An effective neighborhood domain is rectangular when the reference map is built and uses different sizes for each spatial dimension. Nevertheless, by Equation (10), it is clear that an $R^{2}$ neighbour with an area greater than the standard is always verified (Equation (11)).

(11) $$\begin{array}{c} D\;\mathrm{area}=\pi {\left(\mathrm{radius}\cdot \Delta \right)}^{2}\;\;D_{o}\;\mathrm{area}=2\varepsilon_{x}\cdot 2\varepsilon_{y}=4\cdot {\mathrm{radius}}^{2}\cdot \overset{\geq \Delta^{2}}{\overset{⎴}{\Delta x\Delta y}}\\ \mathrm{area}\;\mathrm{ratio}=\frac{D_{o}\;\mathrm{area}}{D\;\mathrm{area}}=\frac{4\cdot {\mathrm{radius}}^{2}\cdot \Delta x\Delta y}{\pi {\left(\mathrm{radius}\cdot \Delta \right)}^{2}}=\frac{4\Delta x\Delta y}{\pi \Delta^{2}}\geq \frac{4}{\pi }>1\; \end{array}$$

### 3.3. Antenna Weighting

RSSI measurements are non-direct physical estimations of a signal state. An RSSI value is obtained after signal decoding through different correlation processes [40,41].

The decoding process achieves data transfer error rate reduction by introducing a high process gain. RSSI measurements can observe high biases for lower signal powers, for which a smart decoding process can achieve a better RSSI in respect to the effectiveness of the signal power. For such cases, in particularly unlucky $\left(x,y\right)$ points, reference map projections can produce RSSI values much lower than the obtained ones.

The bias caused by demodulation process gain is expected to grow in the presence of low signal powers, so an external correction gain can be applied in estimator computing to try to remove this effect and reduce the weight of a weak antenna inside the overall estimator computation. For reduced computational cost algorithms (Equation (4)), the function $F\left(\bar{x}\right)$ can be modified as follows:

(12) $$\begin{array}{cc} C\left(x,y\right)= & F\left(\bar{S}-M\left(x,y\right)\right)=\sum_{i=1}^{M\times N}w_{i}F_{i}\left(\bar{S}-M\left(x,y\right)\right)\\ & \mathrm{with}\;w_{i}=i-\mathrm{th}\;\mathrm{steering}\;\mathrm{vector}\;\mathrm{element}\;\mathrm{related}\;\mathrm{weight}\\ & \;\;\;\;\;\;\;\mathrm{RSSI}\;\mathrm{antenna}\;\mathrm{weighting}\to w_{i}=\frac{10^{\frac{s_{i}}{10}}}{\sum_{n=1}^{M\times N}10^{\frac{s_{n}}{10}}} \end{array}$$

A direct RSSI antenna value can be used to estimate the RSSI estimation reliability, so antenna weighting is applied by placing the weights as shown in Equation (12).

### 3.4. Minimum Variance (minVAR) Estimator

In the localization problem stated in Equation (3), the effective kernel of the localization algorithm is the estimator or pseudospectrum function. An ideal estimator should give a singular minimum point (i.e., an absolute minimum) for the overall localization domain, and it must coincide with the right node position. If ideal hardware is used and localization is required in a perfect environment, in which radio propagation acts perfectly and as modelled in the reference map (Equation (2)), a basic estimator function can be used (Equation (5) [19]).

In [18,19], the maximum accuracy limit is stated performing an analytical Cramer–Rao-bound (CRB) computation [37], as presented in [16], but CRB analysis places only an ideal accuracy limit given by the geometrical distributions of antenna gains over the space. CRB analysis evaluates the minimum achievable error for the localization space, supposing that the only source of idealization is the RSSI AWGN added to the obtained steering vector (modelled by the equivalent σ noise parameter as in [19]).

**Figure 9:**
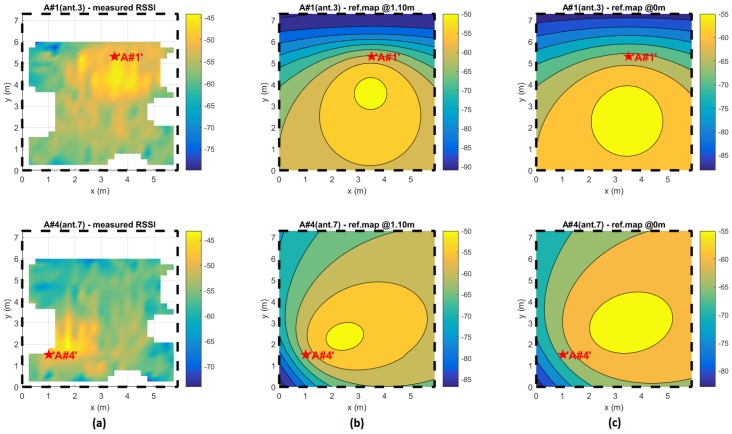
Collected RSSI values over the area (**a**) compared to the estimated RSSI values calculated (**b**) at $z_{REF}=1.10\;m$ and (**c**) at $z_{REF}=0\;m$.

Rather than the measure AWGN, the main source of estimator bias in real applications is the effective inconsistency between the reference map and the effective RSSI distribution over the space. As an example, in Figure 9, the measured RSSI distributions of two different antennas in the experiment site of Figure 1 is shown; next to them, the expected ideal distributions are plotted for two different expected elevations (${z_{\mathrm{ref}}}_{0}$ = 1.1 m and ${z_{\mathrm{ref}}}_{1}$ = 0 m).

Figure 9 clearly shows how much the RSSI distributions differ from the expected projections. In real environments, producing a reliable reference map as proposed in [19,42] is unthinkable without a scenery-related calibration session, as occurs in the fingerprinting approach. Dealing with this, reducing the reference map misalignment effects on localization estimation (causing estimator bias) is required and a new estimator function (respecting Equation (4) definition) is proposed.

(13) $$\begin{array}{cc} & C\left(x,y\right)={\mathrm{var}}_{i}\left\{\bar{S}-M\left(x,y\right)\right\}=E_{i}\left\{{\left[\left(s_{i}-m_{i}\right)-E_{i}\left\{\bar{S}-M\left(x,y\right)\right\}\right]}^{2}\right\}\\ & \begin{array}{c} C\left(x,y\right)=K_{0}\sum_{i=1}^{M\times N}{\left[\left(s_{i}-m_{i}\right)-K_{0}\sum_{j=1}^{M\times N}\left(s_{j}-m_{j}\right)\right]}^{2} \end{array}\;\mathrm{with}\;K_{0}=\frac{1}{M\times N} \end{array}$$

In Equation (13), the minVAR estimator is shown; it evaluates the variance associated with the difference vector built from the measured steering vector and the reference map vector (while the standard least squares estimator calculates its norm using Equation (5)). Assuming a perfect reference map is created, both LSE and minVAR functions act as two unbiased estimators; thus, the CRB of the localization network results, which show the expected accuracy related to typical AWG-noised measures, are the same, as shown in [19].

### 3.5. Fading and Multipath Immunity

A straight evaluation of RSSI parameter intended as an estimation of physical RF received power by the anchor node leads to huge localization estimation errors due to effective RF received power fluctuations due to fading and multipath effects. Dealing with standard and crowded environments such behavior could make the proposed system unusable, but RSSI defined as in IEEE 802.11/802.15.4 network protocols is strictly related to effective data packet information and it is uniquely linked to each different data frame. Data packets are coded through Direct Sequence Spread Spectrum techniques, thus effective data retrieval shows improved immunity towards fading and multipath (representable as delayed receiving signal replies) [43,44,45,46].

**Figure 10:**
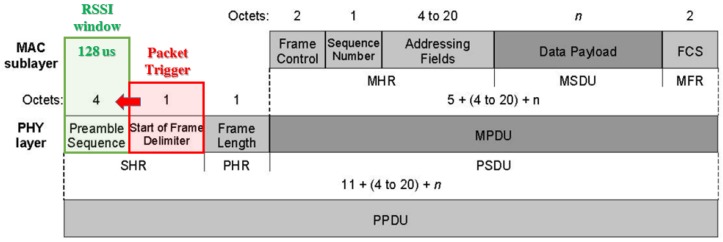
Pictorial of RSSI evaluation scheme within packet frame for IEEE 802.15.4.

As depicted in Figure 10 RSSI estimation is averaged overall the preamble sequence window only after recognizing the packet “Start of Frame Delimiter” through spread spectrum decoding, while spread spectrum correlation techniques ensure that only the first coming packet will be evaluated thus ignoring any delayed echo reply. Furthermore averaging RSSI over the entire preamble sequence window allows to reduce highly variable fading effects on RSSI estimations, while highly destructive effects lead to a packet loss which prevents from obtaining wrong RSSI values that can lead to wrong localization estimations.

For slow fading issues which belong to a constant and directive interference the antenna multiplicity helps to mitigate such phenomena: Two different cases can happen

- omni-directional interference (or rather “diffuse scattering”): any steering vector RSSI value is uniformly altered so the steering vector mean value μ is altered, linearly multiplying the vector for a constant coefficient, but overall linear vector direction remains the same;
- highly directional interference: some vector terms are dramatically altered, but the overall linear values steering vector direction is maintained (only few vector terms ratio are changed).

As highlighted in Section 3 and further in Section 4 and in [17,19,32] the *steering vector* maintains its DoA/positioning information into differences between single RSSI terms (or ratios between linear terms), or rather, into the effective steering vector direction in $C^{M}/R^{M}$ vectorial space.

Having as reference map the ensemble of physically acceptable steering vectors for a given array structure, a good ML algorithm implementation will be able to identify the most similar reliable map vector rejecting/ignoring the extra domain vectorial components.

ML algorithms based on vectorial subspaces decomposition [32,33] evaluates direction match between obtained steering vector and reference vector ignoring at all any constant-term fluctuation while rejecting singular term ratio mismatches. Note that for reduced computational cost subclass algorithms (Equation (4)) such capability depends directly on estimator function.

By this Section 4 will describe how minVAR estimator respect LSE is more able to ignore costant-term mismatches while singular term mismatch effects are minimized increasing the number of distributed antennas, as it will be shown by estimator bias coefficients ratios in (Equations (33)–(35)).

## 4. Estimator Function Improvements Assessment

The effective core of “One-Step” algorithm block relies over the new minVAR estimator function, introduced in Section 3.4. Having to process long vectors of RSSI values that describe a set of distributed antenna gains, Maximum-Likelihood algorithms based on vectorial subspace decomposition (like MuSiC [32,33] or Esprit [47]) become unfeasible due to increase of problem complexity order and for the lack of orthogonality conditions between steering vectors collected from different $\left(x,y\right)$ points. Consequently, the effective estimator function improvements should be evaluated respect to LSE standard estimator implementation, as proposed in Equation (5) [31,35,38], which is at the best of the author knowledge the only suitable estimator.

To achieve estimator bias immunity, a propagation error model for reference map errors in terms of both LSE and minVAR follows.

Considering the reference map bias, the localization problem statement (Equation (3)) is as shown in Equation (14). The equation defines the reference map bias vector object as the RSSI value difference between the ideal projected gain maps and the physically obtained ones (Equation (15)).

(14) $$\underset{\begin{array}{c} \mathrm{biased}\\ \mathrm{estimator} \end{array}}{\underset{⎵}{C\left(x,y\right)}}=F\left(\bar{S}-M\left(x,y\right)\right)\;\Rightarrow \;\underset{\begin{array}{c} \mathrm{unbiased}\\ \mathrm{estimator} \end{array}}{\underset{⎵}{C^{*}\left(x,y\right)}}=F\left(\bar{S}-\left(\underset{\begin{array}{c} \mathrm{physical}\\ \mathrm{reference}\;\mathrm{map}\\ \left(\mathrm{unknown}\right) \end{array}}{\underset{⎵}{\overset{\begin{array}{c} \mathrm{ideal}\\ \mathrm{reference}\;\mathrm{map}\\ \left(\mathrm{computed}\right) \end{array}}{\overset{⎴}{M\left(x,y\right)}}+\overset{\begin{array}{c} \mathrm{bias}\;\mathrm{of}\;\mathrm{ideal}\\ \mathrm{reference}\;\mathrm{map}\\ \left(\mathrm{unknown}\right) \end{array}}{\overset{⎴}{M_{\Delta }\left(x,y\right)}}}}\right)\right)$$

(15) $$\begin{array}{cc} \mathrm{map}\;\mathrm{bias}\;\mathrm{vector}\; & M_{\Delta }\left(x,y\right)={\langle \underset{\mathrm{number}\;\mathrm{of}\;\mathrm{elems}=\sum_{i=1}^{N}M_{i}=M\times N}{\underset{︸}{{m_{\Delta }}_{11}|{m_{\Delta }}_{21}\left|\ldots \right|{m_{\Delta }}_{M_{1}1}|{m_{\Delta }}_{12}\left|\ldots \right|{m_{\Delta }}_{M_{2}2}\left|\ldots \right|{m_{\Delta }}_{1N}\left|\ldots \right|{m_{\Delta }}_{M_{N}N}}}\rangle }^{H}\\ \mathrm{with}\; & {m_{\Delta }}_{ij}\left(x,y\right)=\underset{\begin{array}{c} \mathrm{physical}\;\mathrm{gain}\;\mathrm{projection}\\ \left(\mathrm{unknown}\right) \end{array}}{\underset{⎵}{{m_{ij}}_{\mathrm{PHYSICAL}}\left(x,y\right)}}-\underset{\begin{array}{c} \mathrm{ideal}\;\mathrm{gain}\;\mathrm{projection}\\ \left(\mathrm{calculated}\right) \end{array}}{\underset{⎵}{{m_{ij}}_{\mathrm{IDEAL}}\left(x,y\right)}} \end{array}$$

Following Equation (3), in localization estimation, a formally faultless evaluation of the estimator bias should be made to evaluate how much the reference map bias vector argument can alter the conditions in Equation (9) and shift the position of the minimum pseudospectrum point. Despite this, it must be considered that any consideration about the unknown physical gain map projection trends is totally unfeasible; therefore, its derivatives are undefinable.

The analysis can be simplified with a comparative evaluation of the effects of the bias vector directly to the function image between the different estimators. Without evaluating the $M_{\Delta }\left(x,y\right)$ trend, if an estimator shows a reduced variability in respect to the ${m_{\Delta }}_{i}\left(x,y\right)$ subfunctions, it will be more robust against ideal physical reference map differences. Therefore, a qualitative comparison can be made between the estimator function differences and the ${m_{\Delta }}_{i}$ terms.

The estimator function is directly definable in the $R^{M\times N}$ reference map vectorial space. By this, the estimator gradient can be defined as in Equation (16).

(16) $$\mathrm{map}\;\mathrm{gradient}\;\nabla_{M}C\left(x,y\right)=\langle \frac{\partial C\left(x,y\right)}{\partial m_{1}}|\left.\frac{\partial C\left(x,y\right)}{\partial m_{2}}\right|\ldots |\frac{\partial C\left(x,y\right)}{\partial m_{MN}}\rangle$$

Thus, by defining the gradient vector, each point of the estimator function can be written as in Equation (17), which separates the influence of the reference map bias vector.

(17) $$\underset{\begin{array}{c} \mathrm{computed}\\ \mathrm{estimator}\;\mathrm{value}\\ \left(\mathrm{biased}\right) \end{array}}{\underset{⎵}{C\left(x,y\right)}}\approx \underset{\begin{array}{c} \mathrm{unbiased}\\ \mathrm{estimator}\;\mathrm{value}\\ \left(\mathrm{unknown}\right) \end{array}}{\underset{⎵}{C^{*}\left(x,y\right)}}+\underset{\begin{array}{c} \mathrm{estimator}\;\mathrm{value}\\ \mathrm{alteration}\\ \left(\mathrm{due}\mathrm{to}\mathrm{map}\mathrm{bias}\right) \end{array}}{\underset{⎵}{\overset{\mathrm{estimator}\;\mathrm{bias}\;\mathrm{gain}}{\overset{⎴}{{\left[\nabla_{M}C\left(x,y\right)\right]}_{M\left(x,y\right)}}}\cdot M_{\Delta }\left(x,y\right)}}+\underset{\mathrm{superior}\;\mathrm{order}\;\mathrm{terms}}{\underset{⎵}{O\left({\left|\left|M_{\Delta }\left(x,y\right)\right|\right|}^{2}\right)}}$$

By Equation (17), a brief evaluation of the estimator bias is given by the estimator bias gain (Equation (18)), which is a conceptually approximate map bias to estimator bias gain.

(18) $$C_{B}\left(x,y\right)=\left|\left|{\left[\nabla_{M}C\left(x,y\right)\right]}_{M\left(x,y\right)}\right|\right|$$

As estimator bias gain calculated in a localization point $\left(\hat{x},\hat{y}\right)$ points out how much the specific estimator is susceptible of variation on that point due to map biases, comparison between different estimators bias gains over the overall localization area could identify the more reliable estimator.

However, both LSE (Equation (5)) and minVAR (Equation (13)) estimators are strictly non-linear in terms of their vectorial arguments, so it is necessary to verify whether a first-grade approximation (Equation (17)) is reliable enough.

It has already been said in Section 3.1 that map bias can introduce new relative minimums in different $\left(x,y\right)$ points, and this can happen in map vector domains, as well. Assuming that the map bias is restrained enough to alter the estimator function trend only in the neighborhood of the effective estimation point $\left(\tilde{x},\tilde{y}\right)$, a corollary condition should be that the $M_{\Delta }\left(x,y\right)$ term will not be able to alter the estimator function convexity so that the relative minimum condition (Equation (9)) will still be verified.

Convexity behaviour must be verified using the Hessian matrix (as seen in Equation (9)), but the method is absolutely unfeasible when handling a high dimensional $R^{M\times N}\to R$ function. An alternative way to impose convexity is to evaluate the influence of superior grade terms. It is clear that all non-linear map bias dependency is defined by the residual term in Equation (19).

(19) $$\begin{array}{cc} C_{L}\left(x,y\right) & =\left|O\left({\left|\left|M_{\Delta }\left(x,y\right)\right|\right|}^{2}\right)\right|\\ & \;=\left|C\left(x,y\right)-C^{*}\left(x,y\right)-{\left[\nabla_{M}C\left(x,y\right)\right]}_{M\left(x,y\right)}\cdot M_{\Delta }\left(x,y\right)\right| \end{array}$$

By quantifying non-linear estimators, it becomes possible to foresee the reliability of the estimator bias prediction using the estimator bias gain in Equation (18). To evaluate the most reliable estimator between A and B, both conditions in Equation (20) must be verified; the first one verifies which estimator could be the more stable, while the second condition verifies how much the first condition is reliable.

(20) $$\left\{\begin{array}{c} {\left[C_{B}\left(\hat{x},\hat{y}\right)\right]}_{A}<{\left[C_{B}\left(\hat{x},\hat{y}\right)\right]}_{B}\\ {\left[C_{L}\left(\hat{x},\hat{y}\right)\right]}_{A}<{\left[C_{L}\left(\hat{x},\hat{y}\right)\right]}_{B} \end{array}\right.\;⟺\;A\;\mathrm{more}\;\mathrm{reliable}\;\mathrm{than}\;B$$

Note that each condition must be verified in $\left(\hat{x},\hat{y}\right)$, while the estimator equations directly depend on the collected steering vector $\bar{S}$ and the reference map vector $M\left(\hat{x},\hat{y}\right)$ (Equation (3)). Following the reduced computational cost estimator class definition (Equation (4)), the correct and biased localization estimation is defined below.

$$\begin{array}{cc} \left(\hat{x},\hat{y}\right)={\mathrm{argmin}}_{\left(x,y\right)}\left.C\left(x,y\right)\right.\; & ⟹\;C\left(\hat{x},\hat{y}\right)=F\left(\bar{S}-M\left(\hat{x},\hat{y}\right)\right)=0\\ \left(\tilde{x},\tilde{y}\right)={\mathrm{argmin}}_{\left(x,y\right)}\left.C^{*}\left(x,y\right)\right.\; & ⟹\;C^{*}\left(\tilde{x},\tilde{y}\right)=F\left(\left(\bar{S}-M\left(\tilde{x},\tilde{y}\right)\right)-M_{\Delta }\left(\tilde{x},\tilde{y}\right)\right)=0 \end{array}$$

The estimated localization should be equal to the real position.

(21) $$C^{*}\left(\hat{x},\hat{y}\right)=F\left(\left(\bar{S}-M\left(\hat{x},\hat{y}\right)\right)-M_{\Delta }\left(\hat{x},\hat{y}\right)\right)=0$$

Therefore,

(22) S¯−Mx^,y^=MΔx^,y^+F−1(0)←=0(Equation(4))

Using Equations (21) and (22), each function can be evaluated in the $\left(\hat{x},\hat{y}\right)$ point by writing its dependency from the map bias terms directly, as shown in Equation (23).

(23) $$\underset{(a)\;\mathrm{map}\;\mathrm{bias}\;\mathrm{to}\;\mathrm{estimator}\;\mathrm{bias}\;\mathrm{gain}}{\underset{⎵}{C_{B}=\left|\left|{\left[\nabla_{M}C\left(\hat{x},\hat{y}\right)\right]}_{M\left(\hat{x},\hat{y}\right)}\right|\right|}}\;\;\underset{(b)\;\mathrm{non}-\mathrm{linear}\;\mathrm{estimator}\;\mathrm{factor}}{\underset{⎵}{C_{L}=\left|C\left(\hat{x},\hat{y}\right)-{\left[\nabla_{M}C\left(\hat{x},\hat{y}\right)\right]}_{M\left(x,y\right)}\cdot M_{\Delta }\left(\hat{x},\hat{y}\right)\right|}}$$

### 4.1. Algorithms Based on Vectorial Subspace Decomposition

Estimators based on vectorial subspace projections (e.g., MUSIC [32,33], Esprit [47]) can give very high map bias and noise rejection relying on the property of orthogonality between the error vectors and the expected vectorial subspaces. The bias factor for vectorial subspace algorithms is ideally zero because any $M_{\Delta }\left(x,y\right)$ vector has a null projection over the map vector subspace [32].

Unfortunately, applying vectorial subspace decomposition algorithms on a one-step algorithm is unfeasible due to the high grade complexity of the computation. A fundamental constraint for indoor localization is real-time tracking, but high global steering vector dimensionality (Equation (1)) prevents singular value decomposition and reference map vector projection [32,47] over subspaces within reasonable timeframes. The localization results of RSSI MUSIC implementation (presented in [32]) will be reported to show the mean execution time and speed ratio of each estimator.

### 4.2. LSE Estimator Bias

Applying the condition in Equation (21) to calculate the LSE estimator gradients results in the following equation:

(24) $$C_{\mathrm{LSE}}\left(\hat{x},\hat{y}\right)=\sum_{i=1}^{M\times N}{\left(s_{i}-m_{i}\right)}^{2}=\sum_{i=1}^{M\times N}{{m_{\Delta }}_{i}}^{2}\;\;\;\underset{{\left[\nabla_{M}C_{\mathrm{LSE}}\left(\hat{x},\hat{y}\right)\right]}_{i}}{\underset{⎵}{{\left[\frac{\partial C_{\mathrm{LSE}}\left(x,y\right)}{\partial m_{i}}\right]}_{\left(\hat{x},\hat{y}\right)}}}=-2\left(s_{i}-m_{i}\right)=-2{m_{\Delta }}_{i}$$

Following Equation (23), the estimator bias factors for the LSE estimator are given below.

(25) $$\begin{array}{cc} {C_{B}}_{LSE} & =\sqrt{\sum_{i=1}^{M\times N}{\left({\left[\frac{\partial C_{\mathrm{LSE}}\left(x,y\right)}{\partial m_{i}}\right]}_{\left(\hat{x},\hat{y}\right)}\right)}^{2}}=2\sqrt{\sum_{i=1}^{M\times N}{{m_{\Delta }}_{i}}^{2}}=2\left|\left|M_{\Delta }\left(\hat{x},\hat{y}\right)\right|\right|\\ {C_{L}}_{LSE} & =\left|C\left(\hat{x},\hat{y}\right)-{\left[\nabla_{M}C\left(\hat{x},\hat{y}\right)\right]}_{M\left(x,y\right)}\cdot M_{\Delta }\left(\hat{x},\hat{y}\right)\right|\\ & =\left|\sum_{i=1}^{M\times N}{{m_{\Delta }}_{i}}^{2}-\sum_{i=1}^{M\times N}\left(-2{m_{\Delta }}_{i}\right){m_{\Delta }}_{i}\right|=3\sum_{i=1}^{M\times N}{{m_{\Delta }}_{i}}^{2}=3{\left|\left|M_{\Delta }\left(\hat{x},\hat{y}\right)\right|\right|}^{2} \end{array}$$

### 4.3. minVAR Estimator Bias

By Equation (13), the minVAR estimator is written as follows:

(26) $$C_{\mathrm{minVAR}}\left(x,y\right)=K_{0}\sum_{k=1}^{M\times N}C_{k}\;\;\mathrm{with}\;C_{k}={\left[\left(1-K_{0}\right)\left(s_{k}-m_{k}\right)-K_{0}\sum_{j=1,j\neq k}^{M\times N}\left(s_{j}-m_{j}\right)\right]}^{2}$$

Each term of the minVAR estimator gradient is shown below:

(27) $$\begin{array}{c} \begin{array}{cc} {\left[\nabla_{M}C_{\mathrm{minVAR}}\left(x,y\right)\right]}_{i} & =\frac{\partial C_{\mathrm{minVAR}}\left(x,y\right)}{\partial m_{i}}=K_{0}\sum_{k=1}^{M\times N}\frac{\partial C_{k}}{\partial m_{i}}=K_{0}\left(\frac{\partial C_{i}}{\partial m_{i}}+\sum_{k=1,k\neq i}^{M\times N}\frac{\partial C_{k}}{\partial m_{i}}\right)\\ & \mathrm{with}\;\frac{\partial C_{i}}{\partial m_{i}}=2\left[\left(s_{i}-m_{i}\right)-K_{0}\sum_{j=1}^{M\times N}\left(s_{j}-m_{j}\right)\right]\left(K_{0}-1\right)\\ & \;\;\;\frac{\partial C_{k}}{\partial m_{i}}=2\left[\left(s_{k}-m_{k}\right)-K_{0}\sum_{j=1}^{M\times N}\left(s_{j}-m_{j}\right)\right]K_{0} \end{array} \end{array}$$

Expanding on Equation (27) results in the following equation:

$$\frac{\partial C\left(x,y\right)}{\partial m_{i}}=2K_{0}\left\{\left[\left(s_{i}-m_{i}\right)-K_{0}\sum_{j=1}^{M\times N}\left(s_{j}-m_{j}\right)\right]\left(K_{0}-1\right)+K_{0}\sum_{k=1,k\neq i}^{M\times N}\left[\left(s_{k}-m_{k}\right)-K_{0}\sum_{j=1}^{M\times N}\left(s_{j}-m_{j}\right)\right]\right\}$$

Placing

$$\mu =K_{0}\sum_{j=1}^{M\times N}\left(s_{j}-m_{j}\right)=E_{j}\left\{\bar{S}-M\left(\hat{x},\hat{y}\right)\right\}=E_{j}\left\{M_{\Delta }\left(x,y\right)\right\}$$

it results

$$\frac{\partial C\left(x,y\right)}{\partial m_{i}}=2K_{0}\left\{\left(K_{0}-1\right)\left(s_{i}-m_{i}\right)-\mu \left(K_{0}-1\right)+K_{0}\sum_{k=1,k\neq i}^{M\times N}\left[\left(s_{k}-m_{k}\right)-\mu \right]\right\}=\ldots$$

(28) $$\begin{array}{c} \begin{array}{cc} \;\ldots \; & =2K_{0}\left[\left(K_{0}-1\right)\left(s_{i}-m_{i}\right)-\mu \left(K_{0}-1\right)+K_{0}\sum_{k=1,k\neq i}^{M\times N}\left(s_{k}-m_{k}\right)-K_{0}\left(M\times N-1\right)\mu \right]\\ & =2K_{0}\left[-\left(s_{i}-m_{i}\right)-\mu \left(K_{0}-1\right)+\mu -K_{0}\left(M\times N-1\right)\mu \right]\\ & =2K_{0}\left(-s_{i}+m_{i}-\mu K_{0}+\mu +\mu -\mu +\mu K_{0}\right)\\ & =2K_{0}\left[\mu -\left(s_{i}-m_{i}\right)\right]\;\Rightarrow \;{\left[\frac{\partial C_{\mathrm{minVAR}}\left(x,y\right)}{\partial m_{i}}\right]}_{\left(\hat{x},\hat{y}\right)}=2K_{0}\left(\mu -{m_{\Delta }}_{i}\right) \end{array} \end{array}$$

Following Equation (23), the estimator bias factors for the minVAR estimator are written as follows:

(29) $$\begin{array}{c} \begin{array}{cc} {C_{B}}_{minVAR} & =\sqrt{\sum_{i=1}^{M\times N}{\left({\left[\frac{\partial C\left(x,y\right)}{\partial m_{i}}\right]}_{\left(\hat{x},\hat{y}\right)}\right)}^{2}}=2K_{0}\sqrt{\sum_{i=1}^{M\times N}{\left({m_{\Delta }}_{i}-\mu \right)}^{2}}\\ {C_{L}}_{minVAR} & =\left|\overset{{\mathrm{var}}_{i}\left\{M_{\Delta }\left(\hat{x},\hat{y}\right)\right\}}{\overset{⎴}{{\mathrm{var}}_{i}\left\{\bar{S}-M\left(\hat{x},\hat{y}\right)\right\}}}-2K_{0}\sum_{i=1}^{M\times N}\left(\mu -{m_{\Delta }}_{i}\right){m_{\Delta }}_{i}\right|\\ & =\left|K_{0}\sum_{i=1}^{M\times N}{\left({m_{\Delta }}_{i}-\mu \right)}^{2}-2K_{0}\sum_{i=1}^{M\times N}\left(\mu -{m_{\Delta }}_{i}\right){m_{\Delta }}_{i}\right|\\ & =\left|K_{0}\sum_{i=1}^{M\times N}{{m_{\Delta }}_{i}}^{2}-2K_{0}\mu \sum_{i=1}^{M\times N}{m_{\Delta }}_{i}+K_{0}\left(M\times N\right)\mu^{2}-2K_{0}\mu \sum_{i=1}^{M\times N}{m_{\Delta }}_{i}+2K_{0}\sum_{i=1}^{M\times N}{{m_{\Delta }}_{i}}^{2}\right|\\ & =K_{0}\left|3\sum_{i=1}^{M\times N}{{m_{\Delta }}_{i}}^{2}+\left[\left(M\times N\right)-4\right]\mu^{2}\right| \end{array} \end{array}$$

### 4.4. Estimator Bias Immunity Comparison

In Equation (30), a generic bias model is described. The map bias is given as a Gaussian noise vector distributed over M×N steering vector terms; an eventual $P_{\mathrm{TX}}$ term is embedded into the mean value of the Gaussian noise, because it results to be common to every steering vector component.

(30) $$\begin{array}{c} \begin{array}{c} {\bar{S}}_{collected}=M\left(x,y\right)+M_{\Delta }\left(x,y\right)\;⟺\left\{\begin{array}{c} M_{\Delta }\left(x,y\right)=N_{M\times N}\left(\mu ,\sigma \right)\;\leftarrow \mathrm{overall}\;\mathrm{map}\;\mathrm{bias}\;\mathrm{vector}\\ n_{i}=N_{1}\left(0,\sigma \right)\;\;\leftarrow \mathrm{AWGN}\;\mathrm{variable}\;\mathrm{term}\\ {m_{\Delta }}_{i}=\mu +n_{i}\;\;\;\leftarrow \mathrm{overall}\;\mathrm{map}\;\mathrm{bias}\;\mathrm{term}\\ \mu_{N}=E_{i}\left\{n_{i}\right\}=0\\ \mu =E_{i}\left\{\bar{S}-M\left(x,y\right)\right\}=E_{i}\left\{M_{\Delta }\left(x,y\right)\right\}=P_{\mathrm{TX}}+\mu^{*}\\ \sigma^{2}={\mathrm{var}}_{i}\left\{\bar{S}-M\left(x,y\right)\right\}={\mathrm{var}}_{i}\left\{M_{\Delta }\left(x,y\right)\right\} \end{array}\right. \end{array} \end{array}$$

Applying Equation (25), the LSE bias factor can be calculated as follows:

(31) $$\begin{array}{cc} {C_{B}}_{LSE} & =2\sqrt{\sum_{i=1}^{M\times N}{{m_{\Delta }}_{i}}^{2}}=2\sqrt{\sum_{i=1}^{M\times N}{\left(\mu +n_{i}\right)}^{2}}=2\sqrt{\sum_{i=1}^{M\times N}\mu^{2}+2\mu \sum_{i=1}^{M\times N}n_{i}+\sum_{i=1}^{M\times N}{n_{i}}^{2}}\\ & =2\sqrt{\left(M\times N\right)\mu^{2}+2\left(M\times N\right)\mu_{N}\mu +\left(M\times N\right)\sigma^{2}}\\ & =2\sqrt{M\times N}\sqrt{\mu^{2}+\sigma^{2}} \end{array}$$

(32) $$\begin{array}{cc} {C_{L}}_{LSE} & =3\sum_{i=1}^{M\times N}{{m_{\Delta }}_{i}}^{2}=3\sum_{i=1}^{M\times N}{\left(\mu +n_{i}\right)}^{2}=3\left[\left(M\times N\right)\mu^{2}+2\mu \sum_{i=1}^{M\times N}n_{i}+\sum_{i=1}^{M\times N}{n_{i}}^{2}\right]\\ & =3\left[\left(M\times N\right)\mu^{2}+2\left(M\times N\right)\mu_{N}\mu +\sum_{i=1}^{M\times N}{n_{i}}^{2}\right]\\ & =3\left(M\times N\right)\left(\mu^{2}+\sigma^{2}\right) \end{array}$$

Applying Equation (29), the minVAR bias factor can be calculated as follows:

(33) $$\begin{array}{cc} {C_{B}}_{minVAR} & =2K_{0}\sqrt{\sum_{i=1}^{M\times N}{\left({m_{\Delta }}_{i}-E\right)}^{2}}=2K_{0}\sqrt{\sum_{i=1}^{M\times N}{\left(\mu +n_{i}-E\right)}^{2}}=2K_{0}\sqrt{\sum_{i=1}^{M\times N}{n_{i}}^{2}}=\frac{\sigma }{2\sqrt{M\times N}} \end{array}$$

(34) $$\begin{array}{cc} {C_{L}}_{minVAR} & =K_{0}\left|3\sum_{i=1}^{M\times N}{{m_{\Delta }}_{i}}^{2}+\left[\left(M\times N\right)-4\right]\mu^{2}\right|=K_{0}\left|3\sum_{i=1}^{M\times N}{\left(\mu +n_{i}\right)}^{2}+\left[\left(M\times N\right)-4\right]\mu^{2}\right|\\ & \;\;=K_{0}\left|3\left(M\times N\right)\mu^{2}+6\left(M\times N\right)\mu_{N}\mu +3\sum_{i=1}^{M\times N}{n_{i}}^{2}+\left[\left(M\times N\right)-4\right]\mu^{2}\right|\\ & \;\;=K_{0}\left[3\sum_{i=1}^{M\times N}{n_{i}}^{2}+4\left(M\times N-1\right)\mu^{2}\right]=K_{0}\left[3\left(M\times N\right)\sigma^{2}+4\left(M\times N-1\right)\mu^{2}\right]\\ & \;\;={\left[\sigma^{2}+\frac{4}{3}\left(1-\frac{1}{M\times N}\right)\mu^{2}\right]}_{\left(M\times N\right)>>1}\approx \sigma^{2}+\frac{4}{3}\mu^{2} \end{array}$$

To evaluate the minVAR improvement over the LSE, the following Equation (20) ratio conditions are

$$\left\{\begin{array}{c} {C_{L}}_{minVAR}\left(\hat{x},\hat{y}\right)<{C_{L}}_{LSE}\left(\hat{x},\hat{y}\right)\\ {C_{B}}_{minVAR}\left(\hat{x},\hat{y}\right)<{C_{B}}_{LSE}\left(\hat{x},\hat{y}\right) \end{array}\right.\;\Rightarrow \;\left\{\begin{array}{c} {C_{L}}_{RATIO}>1\\ {C_{B}}_{RATIO}>1 \end{array}\right.$$

By replacing

(35) $$\begin{array}{cc} {C_{B}}_{RATIO} & =\frac{{C_{B}}_{LSE}}{{C_{B}}_{minVAR}}=4\left(M\times N\right)\sqrt{{\left(\frac{\mu }{\sigma }\right)}^{2}+1}\geq 1\;\;\begin{array}{c} \forall \mu ⟺\forall P_{\mathrm{TX}} \end{array}\\ {C_{L}}_{RATIO} & =\frac{{C_{L}}_{LSE}}{{C_{L}}_{minVAR}}=9\left(M\times N\right)\left(\frac{\mu^{2}+\sigma^{2}}{4\mu^{2}+3\sigma^{2}}\right)\geq 1 \end{array}$$

Final conditions in Equation (35) summarize all reliability comparison between minVAR and LSE estimators. When those conditions are verified, minVAR estimator reliability over LSE is proven.

**Figure 11:**
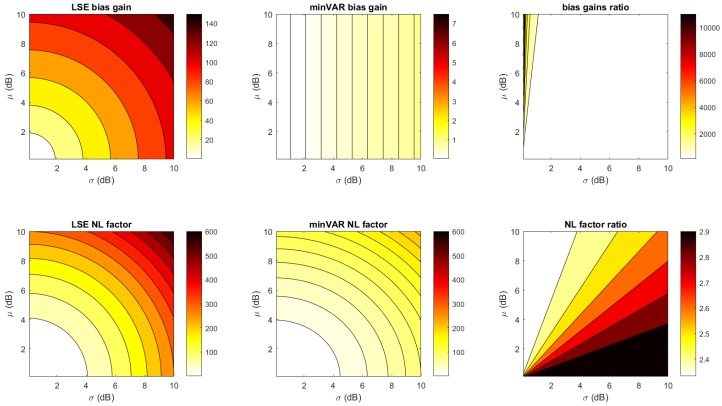
Estimator bias factors for both LSE and minVAR.

In Figure 11, the estimator bias gain factors and estimator factor ratios are shown for the actual experiment configuration (Figure 1 with 4 anchors, each with 7 antennas) with respect to different $\left(\mu ,\sigma \right)$ parameters in the Gaussian map bias model. The estimator factor ratios are always much greater than one, making the minVAR estimator more reliable than the LSE.

By Equation (35), a further noticeable improvement is highlighted: The minVAR estimator is fully independent or unbiased with respect to the user node’s transmitted power term $P_{\mathrm{TX}}$ or rather from each μ steady term (as it can be a $\Delta P_{\mathrm{TX}}$ due to path loss). In particular, if the reference map deviation belongs only to the constant term, then ${C_{B}}_{minVAR}\left(\hat{x},\hat{y}\right)=0$. The improvement is not trivial; every fingerprinting method is dependant on the overall received power value. This dependency causes localization estimation bias for transmitted and received power fluctuations, even though a complete and error-free measured data set is available [19,42].

Note that for minVAR estimator bias gain decreases by increasing the overall number of antennas (Equation (33)) while the estimator non-linearity factor does not change at all (Equation (34)). Therefore, it is proven that minVAR estimator will always enhance its reliability by increasing the number of antennas over the localization space as stated by CRB-analysis [19,37]; LSE estimator instead shows the reverse trend, worsening its bias if each antenna adds its RSSI measurement noise to the steering vector.

Two main cases for map bias distribution can be evaluated: the first considers an highly stable map bias $\left|\mu \right|>>\sigma$ (e.g., for high $\left|P_{\mathrm{TX}}\right|$ terms due to path loss), and the second considers an highly variable map bias (e.g., due to coarse unexpected map model errors).

(36) $$\begin{array}{ccc} \left|\frac{\mu }{\sigma }\right|>>1\; & \Rightarrow \;\left\{\begin{array}{c} {C_{B}}_{RATIO}\approx 4\left(M\times N\right)\left|\frac{\mu }{\sigma }\right|>>1\\ {C_{L}}_{RATIO}\approx \frac{9}{4}\left(M\times N\right)>>1 \end{array}\right. & \Rightarrow \begin{array}{c} \mathrm{minVAR}\;\mathrm{bias}\;<<\;\mathrm{LSE}\;\mathrm{bias} \end{array}\\ \left|\frac{\mu }{\sigma }\right|<<1\; & \Rightarrow \;\left\{\begin{array}{c} {C_{B}}_{RATIO}\approx 4\left(M\times N\right)>>1\\ {C_{L}}_{RATIO}\approx \frac{9}{3}\left(M\times N\right)>>1 \end{array}\right. & \Rightarrow \begin{array}{c} \mathrm{minVAR}\;\mathrm{bias}\;<<\;\mathrm{LSE}\;\mathrm{bias} \end{array} \end{array}$$

### 4.5. Simulated Localization Estimation Results and Comparison

**Figure 12:**
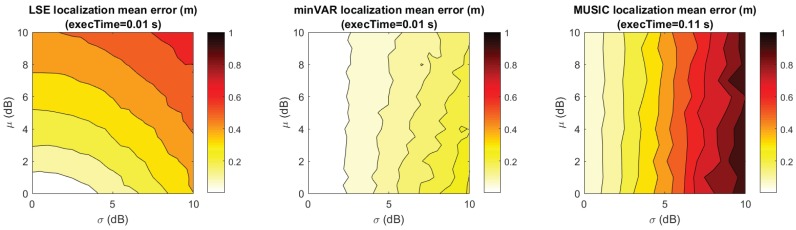
Simulated localization estimations with the Gaussian map bias (mean error).

**Figure 13:**
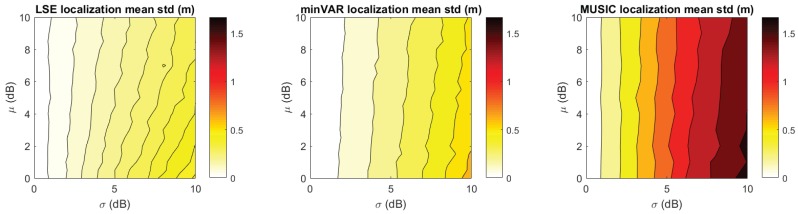
Simulated Localization Estimations With the Gaussian Map Bias (Standard Deviation).

To make an effective comparison, in Figure 12 and Figure 13, the simulated localization error results for the referenced scenery (Figure 1) are shown, computing a set of 10 localizations in each $\left(x,y\right)$ point. For each localization the steering vectors was the corresponding reference map vector $M\left(x,y\right)$ biased with an AWGN map bias defined as in (Equation (30)).

It is remarkable that the plots in Figure 12 follow the predicted trends shown in Figure 11, further highlighting the validity of proposed model. For comparison, Figure 12 and Table 1 show the localization error results of the State-of-Art MUSIC localization algorithm, as shown in [32]. For a 21× slow down in the localization execution time, better localization estimations were achieved.

## 5. Experimental Results

The minVAR estimator can achieve better results than the LSE that are directly comparable to the well-known and high accuracy MUSIC estimator [33,48]. The overall one-step localization algorithm described in Section 3 can be implemented in a known indoor WiFi COTS localization infrastructure. As described in [19] and briefly in Section 1, a network of four IEEE 802.15.4 (as well as IEEE WiFi) compliant anchor nodes (or routers) based on SBA technology [31] (Figure 3) were installed on the ceiling of the office area site, as shown in Figure 1.

The goal is to provide reliable and sufficiently accurate localization without any kind of offline calibration phase, which has been mandatory in the past [1,5,38]. In [19], the novelty has been the replacement of the offline calibration phase with a computed predictive fingerprinting map, but an additional map parameters tuning phase was needed to achieve acceptable results using an LSE-based localization algorithm. Instead in actual experiment the reference map was the straight planar projection of direct angular patch antenna models with parameters as presented in [17] and proposed again in Figure 3: No map tuning phases were performed and the straight ideal map was used.

This work aims to propose an effective implementation of a real-time and calibration-free indoor localization system. To provide a complete characterization of system effectiveness, different kinds of experimental campaigns shall be performed:

- **static localizations**: effective localization accuracy estimation is given through an extensive campaign of localizations achieved throughout the site domain, putting a typical user node on an extensive set of possible positions. Considering a widespread uniform set of positions overall the observation area an highly descriptive error distribution function can be defined, thus characterizing system capability to interact with a non ideal environment which should require specific calibration phases;
- **static localizations with strong scenario changes**: while localizing in a real environment without adding a calibration phase helps to understand how much the localization algorithm is able to overcome “minimal” reference map misalignments, the real complete Plug-and-Play capability is achieved if system functionality is proven also reducing at most any requirement over users deportment (i.e., enabling users to portrait their devices at different heights and with different orientations). As well as a map misalignment is given when an user node is placed at a different height respect reference map $z_{TAG}$ height reference, in the same way using reference maps computed for a $z_{REF}$ different from effective user node height $z_{TAG}$ helps characterizing localization system capability to minimize bias in front of strong utilization scenario changes;
- **dynamic localizations**: the “real-time” localization capability is a specification more linked to the system implementation level than to the effective localization methodology. Despite this, this work aims to show a possible implementation for an effective consumer level Plug-N-Play system: by this, a lifelike example of an actual implementation scenario is given. Different measurements campaign have been done placing a walking user throughout the entire site, making it portraying as a typical standing user holding a standard mobile device;
- **dynamic localizations with strong echo interferer**: to provide an actual demonstration about multipath immunity given by exploiting RSSI measurement through the minVAR estimator (as described in Section 3.5) some additional real-time tracking experiments are done in the presence of a strong echo interferer, thus providing an example of system capability to overcome strong multipath effects.

### 5.1. Static Localizations

**Figure 14:**
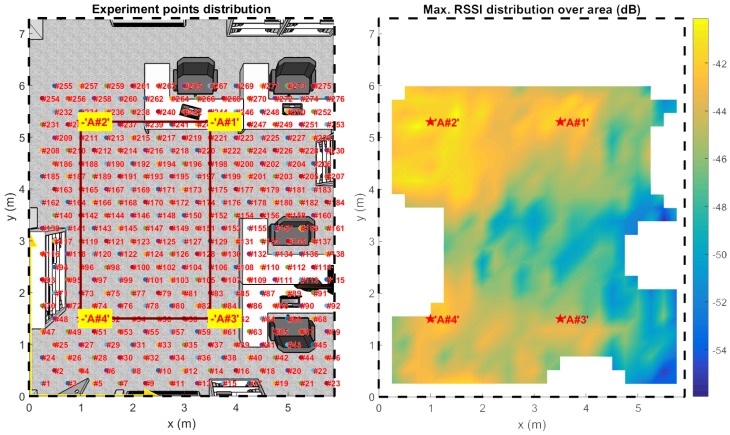
User mobile test-device positions and collected maximum RSSI distribution.

A CP antenna equipped TAG node is to be placed on a grid of known positions (Figure 14) at an equivalent height of about 1.10 m over the floor with anchors pinned on the ceiling at 2.8 m (Figure 1).

**Figure 15:**
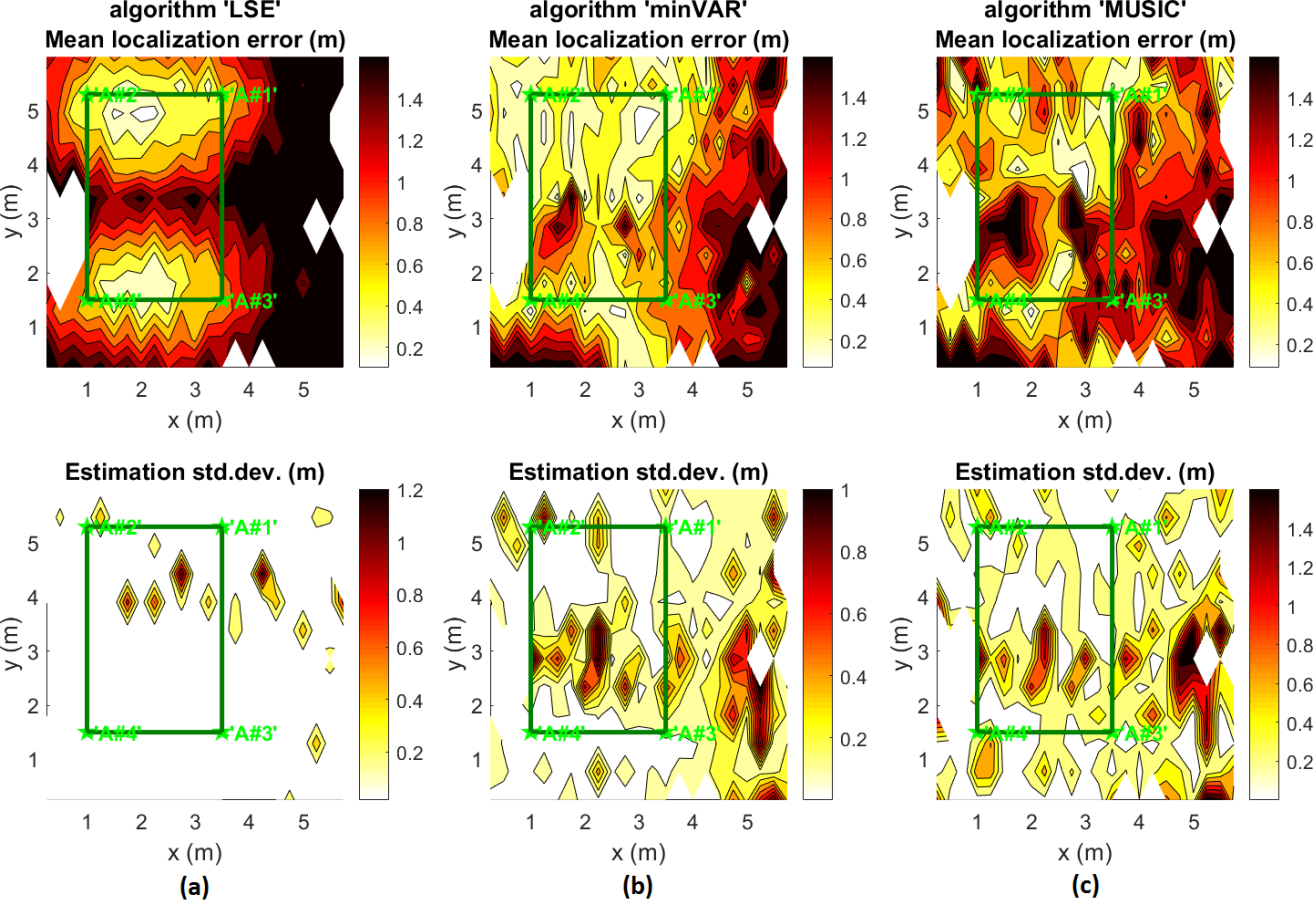
Distributed localization error over $\left(x,y\right)$ with N = 100 trials per point.

In Figure 15, the distribution over $\left(x,y\right)$ of the mean Euclidean localization error and its standard deviation over 100 localization trials for each point are shown. These results were computed using an ideal reference map with pattern projections computed as shown in [19] for a height of 1.10 m from the floor (or rather the exact height for TAG positions).

Figure 15 highlights the accuracy improvement obtained through a raw minVAR estimator implementation, absolutely without enabling the extended features of the preselected area (Section 3.1), adaptive masking (Section 3.2) and antenna weighting (Section 3.3). Figure 15b shows that a raw minVAR implementation is able to achieve a straight 65% area coverage with a sub-metric localization error. The typical LSE algorithm (Figure 15a) implemented without a reference map calibration shows an halved sub-metric error coverage (30%) while the MuSiC implementation depicts a quite unpredictable error distribution (Figure 15c) due to wrong gain vectorial space definition.

Note that only 36% of all the experiment points belong inside the mesh area (shown as a bounding box in Figure 14). The mesh was built to have a limited coverage of the overall site area to demonstrate the capability of the localization system to work where a high dilution of precision is expected. While Figure 15 offers an introductive performance comparison, more detailed comparisons follow. The overall results are evaluated for the mesh area only and for the complete site area, placing a direct comparison with results of [19] and an indirect comparison with other State-of-Art indoor positioning systems in the existing literature.

**Figure 16:**
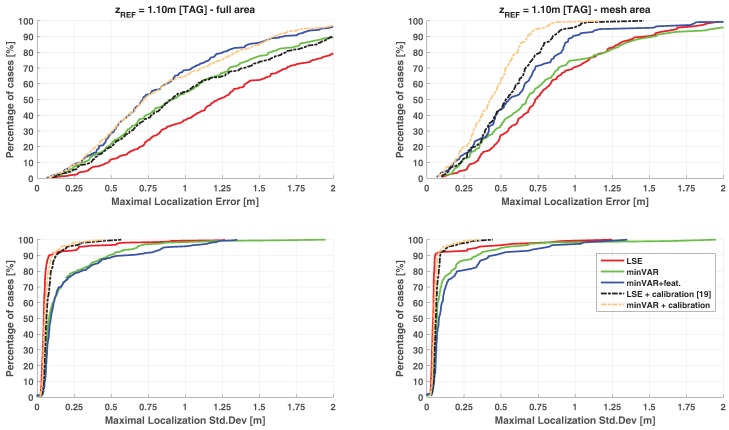
Cumulative distribution functions (CDFs) using the reference map @$z_{REF}=1.10m=z_{TAG}$.

In Figure 16, the cumulative distribution functions (CDFs) of the localization error are shown applying the entire “One-Step” algorithm block implementing the LSE and minVAR algorithms. The final “One-Step” implementation corresponds to the “minVAR+features” trace, specifying the enabling of all the proposed extended features (or rather, the preselected area (Section 3.1), adaptive masking (Section 3.2) and antenna weighting (Section 3.3)).

An important achievement for this work is the capability of obtaining a sub-metric localization accuracy without the needing of the calibration phase: The dashed lines in Figure 16 describes localization error results obtained by [19] applying a raw LSE algorithm using a calibrated reference map.

In [19], the reference map was given as a parametrical function over $\left(x,y\right)$ with a set of angular parameters regarding each antenna of the SBAs (i.e., the HPBW angles, the expected front-to-back ratio and the ratios of the different antenna gains) plus an α parameter corresponding to the η free-space path loss exponential of the Friis transmission equation [19]. Localization results of [19] have been obtained through a complex parametric tuning of parameters described above, applying some manual corrections after observation of overall RSSI measurements.

From Figure 16, it is evident that results obtained through minVAR estimator using a totally uncalibrated (or rather, purely ideal) reference map are directly comparable with the results of the raw LSE implementation using the carefully calibrated map. Enabling the extended features actual results are far better than the LSE calibrated one: note that in actual implementation the calibration routine is still applicable, but it is interesting to show that effective reference map error reduction achieved through calibration (Figure 16 “minVAR+calibration” trace) is almost useless thanks to minVAR bias immunity improvement.

#### Static Localizations with Strong Scenario Changes

**Figure 17:**
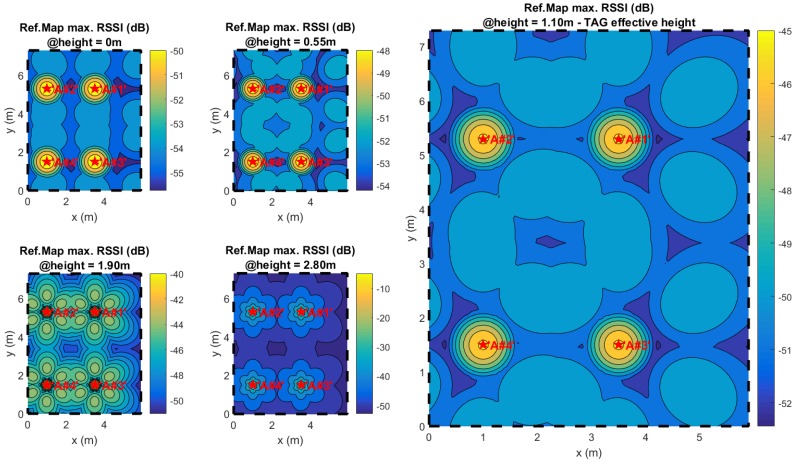
Applied reference maps computed for different heights; TAG height = 1.10 m.

A trivial method to verify the localization estimation immunity over the map bias is to make the localization estimations using a reference map computed for a TAG height different from the effective one. Figure 17 shows how the $z_{REF}$ height projection parameter alters the map projection considerably. Looking over the projection operation shown in [19], it is clear that given a fixed point $\left(\tilde{x},\tilde{y}\right)$ TAG height variations will lead to highly different global steering vectors due to different distance variations between each antenna and the TAG node.

Note that by Equations (2) and (14) global steering vector bias can be considered equivalent to a specific map bias vector added to the reference map in the $\left(\tilde{x},\tilde{y}\right)$ point. Therefore, computing localization using reference maps computed for different heights will be equivalent to obtain a global steering vector bias due to height variation.

Different localization sessions are conducted for the entire set of experiment points using different reference maps calculated for various heights (0 m, 0.55 m, 1.9 m and 2.8 m; Figure 17), which are different from the effective TAG height. Following map bias effect reciprocity as stated above, the computed localization results are able to show estimator stability with respect to vertical TAG motion, placing that the global steering vector variation is due only to geometrical projections differences.

**Figure 18:**
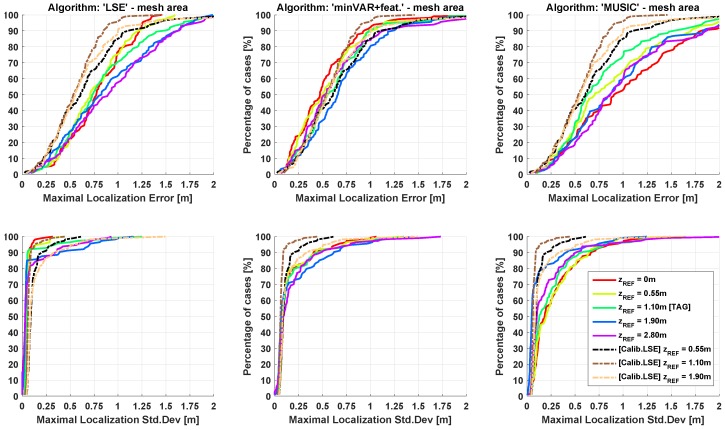
CDFs using different $z_{REF}$ reference maps (mesh area).

**Figure 19:**
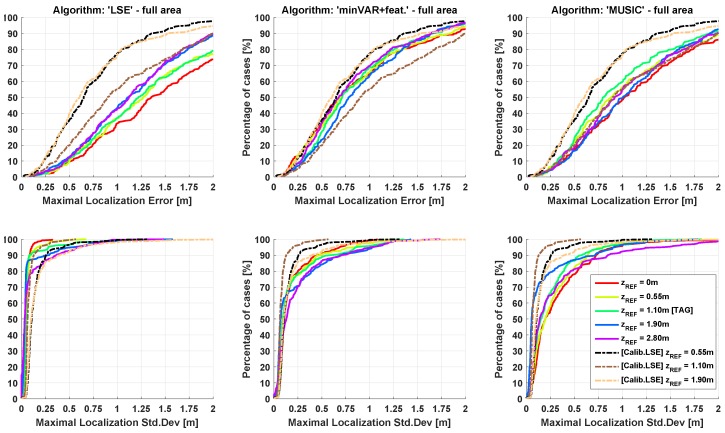
CDFs using different $z_{REF}$ reference maps (full area).

In Figure 18 and Figure 19, the resulting CDFs for the same experimental data set are given, each for a different reference map. The estimation stability in respect to height variations is perceivable through CDF dispersion; a lower bias immunity corresponds to a higher variance over the mean error and error parameters.

The dashed lines show the effect of an overfitting map parameter, as in [19]. It is worthwhile to highlight that, while parameter calibration helps the standard LSE algorithm perform better when the reference map height is equal to the TAG height, the difference between the CDF traces for different $z_{REF}$ parameters worsen.

When the considered localization area is limited to the mesh area, the calibrated reference maps are able to bind the accuracy variance below the uncalibrated LSE execution; uncalibrated minVAR with features gives directly comparable results. Extending the localization area to the overall site, the calibration shows remarkable accuracy gain in respect to the LSE algorithm, while uncalibrated minVAR shows a comparable overall accuracy with much less variation over $\Delta z_{REF}$.

Table 2 and Table 3 summarize the overall localization estimation results. Defining the coverage as the percent of the interested area points with submetrical localization error, the coverage variance column briefly describes the overall dispersion between the CDFs. Overall localization error parameters are evaluated for the estimations calculated using the right $z_{REF}=z_{TAG}$ reference map.

As depicted by Table 2 and Table 3, the proposed approach is capable of ensuring remarkable localization accuracy compared to the LSE best-fit calibration without any kind of calibration at all. Obviously, as can be seen in Figure 16, the calibration routines are still applicable to further improve the localization accuracy results without the high sensitivity to experiment parameters variations, which can worsen the standard algorithms.

### 5.2. Dynamic Localizations

Static localization results describe an acceptable systematic sub-metrical accuracy over the entire 35 m^2^ office area. Localization error distribution as shown in Figure 15 appears to be quite regular and estimations computed through different heights calculated reference maps have been demonstrated to maintain the same error distribution applying minVAR estimator function (Figure 18 and Figure 19).

Fading and multipath phenomena effects can appear heavily reduced if localization tests are done only on a set of static positions. Within a standard static environment multipath paths could be quite static and predictable throughout the site area thus a static test is not enough to argue that actual system is sufficiently immune to fading effects (i.e., Figure 15 higher localization error distribution could depend on static fading interferences); in addition to this, fast fading effects are absent altogether if the site is clear and the testing user device is in a stationary position.

**Figure 20:**
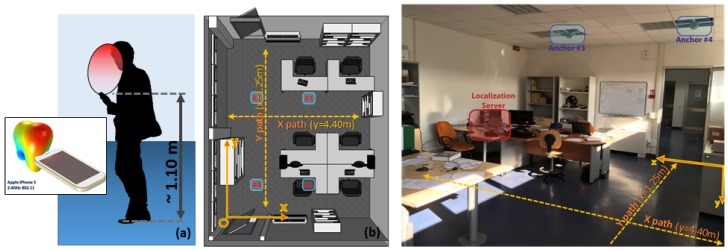
Common standing user posture (**a**) and tracking experiment site and paths (**b**).

To exploit effective fading immunity some tracking tests are performed. Such tests assume the observation of one or more user nodes which are keep in motion by their users walking within the area: thinking at a standard WiFi PIFA installed on a typical smartphone (Figure 20a), the testing 802.15.4 user nodes are equipped with a similar pattern antenna oriented towards the ceiling.

Tracking tests are performed having a walking user (moving at about 5 km/h) throughout the office site following paths highlighted in Figure 20b, handling its device as in Figure 20a.

Tracking sampling rate is related only to coarse communication required time: for a single user node a valid *global steering vector* is obtained when an estimated RSSI is obtained for each antenna of the global anchor network SBAs. Proposed communication scheme suppose to perform a network-user node communication having only one transmitting anchor for each communication while the others can sniff overall network packets (because all the anchors act as a single distributed 802.15.4/802.11 router): for the proposed system each anchor obtains its part of the global steering vector when it completes an RSSI collection cycle on its own antennas.

An RSSI collection cycle is completed when for each antenna of a single SBA a network packet is sent by the user node: the actual 802.15.4 implementation provides a data transfer rate of about 250 kbit/s with a packet length of 60 bytes. Thus global steering vector sampling rate is equal to

(37) $$\begin{array}{c} \begin{array}{cc} T_{s}= & \left(M\times N\right)\times \left(\frac{\mathrm{packet}\;\mathrm{length}\;[\mathrm{bytes}]}{\mathrm{datarate}\;[\mathrm{bytes}/s]}\right)\\ = & \left(M\times N\right)\times \left[{\left(\frac{250\cdot 10^{3}}{8}\;\mathrm{kBytes}/s\right)}^{-1}\cdot 60\;\mathrm{bytes}\right]\approx 28\times 1.9\;\mathrm{ms}=53.2\;\mathrm{ms}\\ & \mathrm{with}\;\left(M\times N\right)=\;\mathrm{number}\;\mathrm{of}\;\mathrm{distributed}\;\mathrm{antennas} \end{array} \end{array}$$

Final localization sampling time must include the algorithm computing time. Following results in Table 1 for actual sub-optimal MATLAB implementation, global sampling time results equal to

(38) $$T_{obs}=T_{s}+T_{alg}=53.2\;\mathrm{ms}+10\;\mathrm{ms}\approx 63.2\;\mathrm{ms}$$

Considering a typical walking speed of about 5 km/h, for a walking user the effective spatial sampling rate becomes

$$\Delta s=T_{obs}\cdot \left(5\cdot \frac{10}{36}\;m/s\right)\approx 8.8\;\mathrm{cm}$$

thus indicating that proposed system is feasible for real-time tracking.

**Figure 21:**
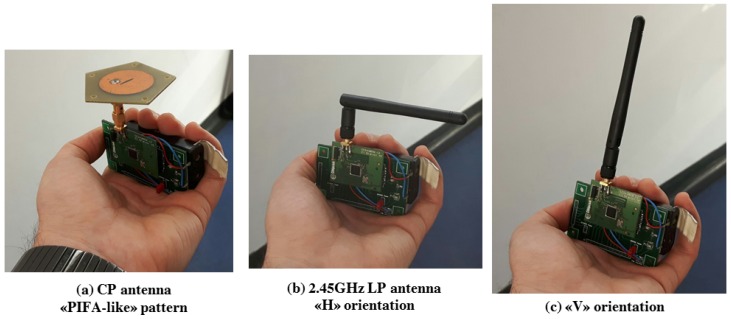
Tracking tests different antenna configurations.

Tracking paths are thought to put the user in “close” areas surrounded by furnishings as various metallic desks (Figure 20b center and right) and some wooden shelves (Figure 20b left). The device is hold at a typical 1.10 m device height for a loose walking user (Figure 20a): for each chosen path different trials are performed using different kinds of antenna on the user device (Figure 21).

The best match for the “smartphone” use case in Figure 20a is given by the CP antenna configuration but to maximize fading observation and to highlight system strength among different user device antenna types, tracking tests are done also for two generic 2.45 GHz dipole LP antenna cases (Figure 21b,c). Using LP antennas, the multipath rejection aid given by CP polarization will be absent in all cases.

**Figure 22:**
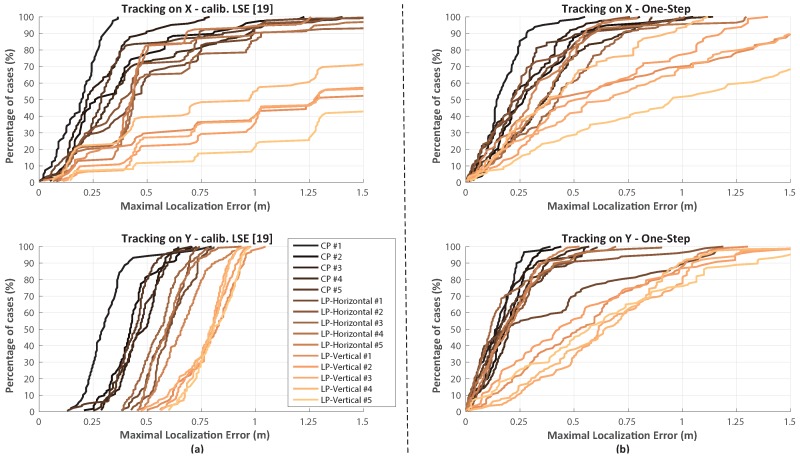
Tracking Error Results CDFs using calibrated LSE [19] (**a**) and uncalibrated One-Step (**b**).

A detailed tracking error analysis is given by overall error Cumulative Distribution Functions proposed below for each tracking test achieved, for both the preceding LSE tuned implementation as in [19] (Figure 22a) and for the actual proposed one (Figure 22b). Table 4 and Table 5 summarize the overall results.

**Figure 23:**
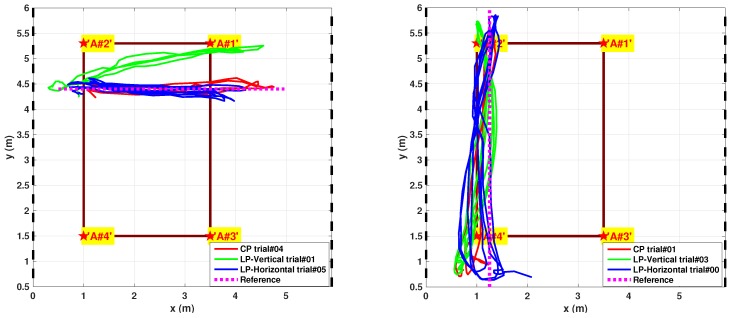
Best performing tracking results for X path (**Left**) and Y path (**Right**).

Figure 23 depicts tracking results respect proposed reference paths. The CP antenna case is the one which better models a typical smartphone PIFA device: Nevertheless the LP cases allow to estimate system behavior dealing with a fading impaired environment.

Overall results clearly show an accuracy worsening for LP vertical oriented dipole case (Figure 21c). Such behavior is explainable due to exactly vertical dipole radiation pattern inversion, for which the LP dipole shows an equivalent isotropic pattern over the XY plane (thus confusing mesh anchor proximity estimation) while within close proximity effective received power is highly reduced.

Nevertheless, the “One-Step” solution still performs far better than previous calibrated LSE implementation [19]. Regarding fading issues X-path tracking trials are more representative due to furnishings arrangement (Figure 20): for the worst cases (LP cases on X-path) the “One-Step” implementation clearly shows an high accuracy improvement.

#### Dynamic Localizations with Strong Echo Interferer

As suggested in Section 3.5, localization estimation done using power-only measurements can be highly affected by fading and multipath phenomena. Actual IEEE 802.15.4/802.11 stacks implement advanced protocol techniques to overcome such effects reducing their disruptive action over packet data transmission [43,44,45,46]; in the same way, antenna input power measurements are post-processed before obtaining effective RSSI values.

As briefly described in Section 3.5, a coarse RSSI processing is able to overcome estimation fluctuations due to highly variable effects (the “fast fading”) by averaging power measurement over a window. By this, final RSSI linear estimation shows a value shift related to the “slow fading” effect only which can be described in terms of physical spreading over the entire set of distributed antennas.

Considering the possible interferer spreading behavior as classified in Section 3.5, a really rough model for slow fading effect over steering vector observation can be modeled using the generic model of reference map bias described in Equation (30). Applying a first grade approximation, slow fading effect could be brought back to an equivalent map bias vector featuring an unknown mean value and a low probabilistic dispersion throughout the different antennas (i.e., a low σ parameter for Equations (30)–(35).

Theoretically the minVAR estimator is able to overcome such kind of map biases, so actual localization should be highly immune to multipath effects.

**Figure 24:**
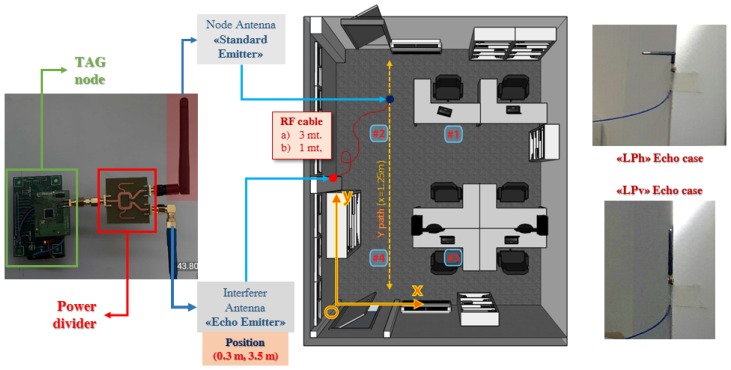
Strong Echo Interferer Experiment Configuration: Node structure (**Left**) experiment site (**Right**).

Some experiments are done to validate such models. A possible way to impose a bad multipath condition is to put within the site a strong interferer distributed as it can be expected by a coarse first grade RF reflection model. Following such criteria some tracking tests are done using the experimental TAG node (Figure 21 and Figure 24) putting on the antenna RF connector a Wilkinson power splitter, connecting both a linear 2.45 GHz dipole for voluntary TAG transmission and an additional 2.45 GHz dipole through an RF cable to act as a strong interferer (Figure 24).

Experiments are repeated for two different RF cable lengths, and for each test the interferer antenna is placed both in an equivalent horizontal polarization respect the anchor network (Figure 24—“LPh” Echo Case) and in an equivalent vertical polarization (Figure 24—“LPv” Echo Case) to model the two main cases of reflection.

Dealing with 2.45 GHz signals effective signal delays are totally negligible respect RSSI window averaging length (128 us for 802.15.4, Figure 10) thus effective interferer immunity can be related to algorithmic improvements.

The interferer is placed adherent to the left wall of the site (at $x_{I}$ = 0.30 m, $y_{I}$ = 3.5 m—Figure 24) thus enforcing the interfering action by profiting of equivalent RF signal reflection given by the wall itself. Because a symmetric power divider is used, effective transmitted power at the input of the mobile antenna (the 2.45 GHz linear dipole) and at the input of the interferer path (composed by a “long” RF cable connected to a second identical 2.45 GHz dipole) is absolutely the same. Effective ratio between desired transmitted signal and interferer (or rather an equivalent SIR—Signal-to-Interferer Ratio) is given only by RF cable losses: Table 6 lists actual SIRs for both RF cables applied.

A set of 50 localization is computed connecting a 50 Ohm termination on the mobile antenna connector to validate interferer operation: Table 7 shows overall mean localization error and standard deviation for each different case. Direct interferer antenna radiation pattern is dramatically altered by the wall (acting as an adherent ground plane) thus interferer localizations are quite noised, but localization error parameters are still under 1.5 m (or rather considerable “metrical”).

Results in Table 7 demonstrate that the interfering antenna is an effective signal source. Average RSSIs collected are absolutely within the typical range of RSSIs for a typical node within the site area (as it can be seen in Figure 9) so putting an identical linear dipole on the mobile antenna connector clearly produce a situation where two identical and identifiable signals are present.

In Table 7 two columns are highlighted: Such columns lists the equivalent parameters which directly feeds the observation/reference map bias model as proposed in Equations (30)–(35) of Section 4.4. Proposed values are extracted evaluating the statistical distribution of RSSI values throughout every single collected steering vector, then the parameters obtained for each steering vector have been averaged throughout the entire set of observations. It is worthwhile to point out how the interferer only observations feature a low σ/μ ratio, due to particular signal spreading case following a “diffuse scattering” propagation model.

Due to RSSI estimation path as depicted in Section 3.5 it has been shown that each diffuse scattering phenomena can be brought back to an highly spreaded RSSI values alteration on collected steering vectors. Following estimator functions theory depicted in Section 4 the minVAR estimator should be more able than LSE to overcome such observation bias, thus tracking experiments should prove this.

**Figure 25:**
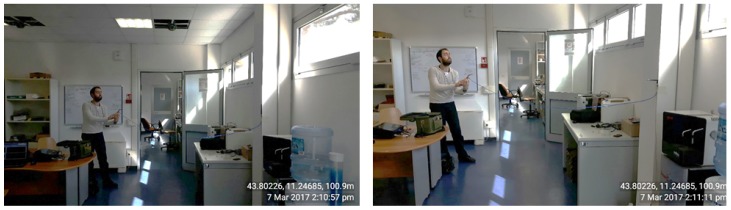
Experimental Session photo: LPv Case (**Left**) LPh Case (**Right**).

Overall tracking error is evaluated moving the node throughout the “Y-path” already described in Section 5.2 and shown in Figure 24. The choice of the “Y-path” is merely due to scenario constraints, because effective tracking experiments are done by moving the mobile node carrying also the RF cable for interferer antenna feeding (Figure 25).

**Figure 26:**
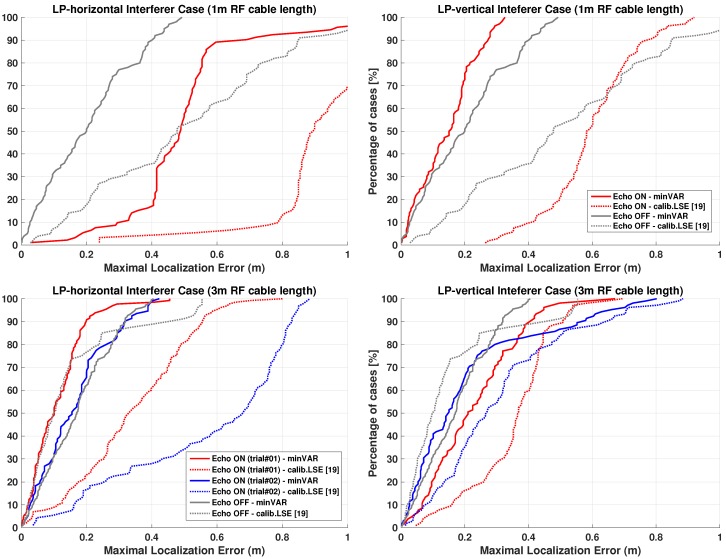
Tracking Error Results CDFs for all test cases: 1 m RF cable (**Above**) and 3 m RF cable (**Bottom**).

Overall error distribution functions are plotted in Figure 26 while Table 8 summarizes most significant results. Table 8 clearly depicts how uncalibrated One-Step applying the minVAR estimator is able to better overcome steering vectors alterations introduced by the interferer, while results for both LSE and for minVAR shows that applying an advanced protocol scheme incidence of fading problems is widely reduced.

In fact localization results obtained using the old calibrated LSE [19] does not show a dramatic impact of interferer, but the minVAR estimator function result to be more reliable as predicted by model described in Section 4 applying phenomena parameters as identified in Table 7.

## 6. System Validation and “State-of-Art” comparison

The applied reference tuned map model projection seen in [19] gives a first-grade approximation of RSSI environment distribution without evaluating any advanced effects, such as fading or scattering [50] or measurement device deviation [51,52], which dramatically alters the effective power distribution [8]. Due to the unpredictability of such phenomena, minimizing the effects of non-idealities will be much more reliable than making any kind of calibration, giving the unknown dependency and environment variables. Some solutions are proposed to reduce fingerprint map errors [53], but the given improvement is far from achieving acceptable accuracy results. The depicted analysis and experimental results show an improved robustness of the proposed system to allow for the implementation of host-side software based solutions.

Table 9 lists a comparison of the State-of-Art equivalent indoor localization systems in the literature, considering standard COTS systems based on IEEE 802.11- and 802.15.4-compliant networks only, as the main goal is to produce a new direct-to-use submetrical localization system that can be considered transparent and cost-free in terms of a standard WiFi network configuration [5]. To provide a comparison with systems based on more refined CSI (Channel State Indicator) evaluations ignoring the loss of portability due to unavailability of CSI detection (available only for OFDM WiFi modulation schemes [54]) on wide-spread IEEE WiFi protocols, also results given by the state-of-art CSI-based localization system presented in [54] are cited.

In literature some accurate indoor localization systems based on TDoA method over 802.11 IEEE networks are presented [6,7] but such method is currently unfeasible using COTS due to high accuracy measurement devices required or the needing of low-level communication stack access.

Explicitly smartphone-related algorithms (i.e., all odometry-based algorithms of Table 9) improve localization accuracy by accomplishing RSSI fingerprint localization with odometric information provided by smartphone sensors. Some papers [15] present approaches not based on fingerprinting techniques; nevertheless, all of them rely on an offline training phase. Although the accuracy can be good, the training phase always requires an uncomfortable calibration session.

## 7. Conclusions

The advantage given by the proposed solution is not trivial at all. System accuracy in bigger environments can be achieved using less anchor nodes in LOS sites (e.g., a single room) while for non LOS sites (e.g., between different rooms) the number of anchors may have to be increased, so the direct comparison of anchor node density (specifically in comparison with [15,58]) is at risk of being misleading. Good wireless network distribution planning tends to maximize the number of anchors inside more complex non LOS areas to maximize connected users’ quality of service; therefore, the necessary conditions allow the proposed localization solution to have good accuracy. The localization service can be provided both through a centralized server or through the user host because steering vectors can be obtained directly from the network of anchors without any network overhead at all. The overall accuracy will not depend on odometric information accuracy, which is dependent from each user node hardware and there is no longer the needing for a tracking filtering.

The overall presented localization solution is thought to be a plug-and-play system without an offline calibration phase to achieve an acceptable localization accuracy. Furthermore, the system is self-consistent and does not require any kind of data fusion with additional sensor information (different from typical fused RSSI and odometric indoor localization with tracking approaches [15,56,57,58]). Because the hardware is based on standard 802.11 and 802.15.4 anchor nodes with the only hardware improvement being the use of a switched-beam array as the antenna, the localization service is given as additional and transparent, using standard network operations; thus, the network administrator is given the ability to provide indoor localization and data contextualization directly to users using only standard network configuration procedures.

Such solution is considerable “infrastructure-free” respect the user experience because any modification to user device nodes is not necessary: as a matter of fact, the system totally shares the existent topology of typical 802.15.4-802.11 LAN networks, and the anchor nodes substitution will not change pre-existent wireless network functionality. Obviously an effective ad-hoc infrastructure must be installed, but its installation concerns only the initial wireless network planning and setup thus assuring to every user the straightaway access to the localization service accessing it directly through the application layer.

## Figures and Tables

**Table 1:** Simulated localization estimations of all the cases (N = 10 localizations for each $\left(x,y\right)$ point).

Estimator	Mean Error (m)	Mean Std.Dev. (m)	Execution Time (ms)	Speed Ratio
LSE	0.37 m	0.20 m	10 ms	21×
minVAR	0.11 m	0.26 m	10 ms	21×
MUSIC	0.47 m	0.87 m	210 ms	1×

**Table 2:** Localization Estimation Result Comparison (Mesh Area [10 m^2^]) @$z_{REF}$ = 1.10 m.

Method	Mean Error (m)	Mean Std.Dev. (m)	Coverage (%) with Error < 1.0 m	Coverage Variance (%) Overall Δz*_REF_*
LSE + calib. [19]	0.55 m	0.07 m	93%	$\left(96\%-85\%\right)=11\%$
LSE	0.83 m	0.08 m	71%	$\left(80\%-58\%\right)=22\%$
LSE + feat.	0.53 m	0.15 m	80%	$\left(95\%-67\%\right)=28\%$
minVAR	0.62 m	0.09 m	75%	$\left(91\%-75\%\right)=16\%$
minVAR + feat.	0.60 m	0.19 m	92%	$\left(92\%-82\%\right)=10\%$
MUSIC	0.81 m	0.26 m	75%	$\left(75\%-53\%\right)=22\%$
MUSIC + feat.	0.78 m	0.25 m	77%	$\left(77\%-60\%\right)=17\%$

**Table 3:** Localization Estimation Result Comparison (Overall Area [35 m^2^]) @$z_{REF}$ = 1.10 m.

Method	Mean Error (m)	Mean Std.Dev. (m)	Coverage (%) with Error < 1.0 m	Coverage Variance (%) Overall Δz*_REF_*
LSE + calib. [19]	1.08 m	0.08 m	55%	$\left(80\%-55\%\right)=25\%$
LSE	1.37 m	0.08 m	38%	$\left(45\%-33\%\right)=12\%$
LSE + feat.	1.27 m	0.15 m	42%	$\left(62\%-40\%\right)=22\%$
minVAR	1.08 m	0.19 m	58%	$\left(61\%-47\%\right)=14\%$
minVAR + feat.	0.87 m	0.21 m	70%	$\left(70\%-65\%\right)=5\%$
MUSIC	1.00 m	0.26 m	61%	$\left(61\%-48\%\right)=13\%$
MUSIC + feat.	0.98 m	0.29 m	62%	$\left(62\%-50\%\right)=12\%$

**Table 4:** X-path Tracking estimation error results @$z_{REF}$ = 1.10 m.

Case		Calibrated LSE [19]		Uncalibrated One-Step	
		Mean Error (m)	Coverage (%) with Error < 1.0 m	Mean Error (m)	Coverage (%) with Error < 1.0 m
**CP** $\begin{array}{c} \left(\mathrm{less}\;\mathrm{fading}\right) \end{array}$	#1	0.18 m	100%	0.16 m	100%
	#2	0.36 m	96.15%	0.31 m	98.72%
	#3	0.28 m	100%	0.26 m	100%
	#4	0.43 m	93.06%	0.38 m	97.69%
	#5	0.36 m	94.26%	0.28 m	100%
	#1	0.49 m	92.47%	0.30 m	100%
	#2	0.45 m	92.31%	0.26 m	100%
**LP-H**	#3	0.62 m	85.23%	0.43 m	98.66%
	#4	0.50 m	89.47%	0.38 m	94.74%
	#5	0.46 m	94.56%	0.31 m	100%
**LP-V** $\begin{array}{c} \left(\mathrm{worst}\;\mathrm{fading}\right) \end{array}$	#1	1.22 m	39.77%	0.66 m	69.32%
	#2	1.28 m	35.36%	0.61 m	76.83%
	#3	1.23 m	40.16%	0.74 m	68.85%
	#4	0.95 m	54.54%	0.44 m	92.92%
	#5	1.46 m	20.93%	1.06 m	51.16%

**Table 5:** Y-path Tracking estimation error results @$z_{REF}$ = 1.10 m.

Case		Calibrated LSE [19]		Uncalibrated One-Step	
		Mean Error (m)	Coverage (%) with Error < 1.0 m	Mean Error (m)	Coverage (%) with Error < 1.0 m
**CP**$\begin{array}{c} \left(\mathrm{less}\;\mathrm{fading}\right) \end{array}$	#1	0.31 m	100%	0.15 m	100%
	#2	0.43 m	100%	0.18 m	100%
	#3	0.47 m	100%	0.17 m	100%
	#4	0.46 m	100%	0.21 m	100%
	#5	0.43 m	100%	0.20 m	100%
**LP-H**	#1	0.60 m	100%	0.37 m	88.98%
	#2	0.60 m	100%	0.25 m	96%
	#3	0.56 m	100%	0.17 m	100%
	#4	0.60 m	100%	0.20 m	100%
	#5	0.66 m	100%	0.20 m	100%
**LP-V**$\begin{array}{c} \left(\mathrm{worst}\;\mathrm{fading}\right) \end{array}$	#1	0.80 m	87.70%	0.54 m	97.54%
	#2	0.79 m	86.73%	0.53 m	100%
	#3	0.76 m	76.98%	0.67 m	100%
	#4	0.78 m	89.92%	0.63 m	100%
	#5	0.81 m	75.21%	0.64 m	100%

**Table 6:** Experimental SIRs obtained through applied RF cables.

Cable Type	$\begin{array}{c} \mathrm{Cable}\mathrm{Attenuation}\\ (\mathrm{dB}/m)@2.45\mathrm{GHz} \end{array}$	Length	$\begin{array}{c} \mathrm{Equivalent}\mathrm{SIR}\\ \left(\mathrm{dB}\right) \end{array}$
RG316-DS [49]	1.33	1 m	1.33
		3 m	4

**Table 7:** Static interferer localization results over 50 trials.

Interferer Polarization Case	Cable Length (m)	Mean Error (m)	Standard dev. (m)	S.Vector Mean Average (dB) *μ* in Equations 30–35	S.Vector Mean std.dev. (dB)	S.Vector std.dev. Average (dB) *σ* in Equations 30–35
**LP-horizontal**	1 m	0.80 m	0.68 m	−62.69	0.85	7.65
	3 m	1.21 m	0.07 m	−65.23	0.37	7.30
**LP-vertical**	1 m	1.01 m	0.24 m	−59.72	0.61	6.64
	3 m	1.04 m	0.42 m	−63.79	1.19	6.92

**Table 8:** Overall Tracking Y-path estimation errors with interferer.

Interferer Case		Calibrated LSE [19]		Uncalibrated One-Step	
		Mean Error (m)	Coverage (%) with Error < 0.5 m	Mean Error (m)	Coverage (%) with Error < 0.5 m
	1 m, SIR = 1.3 dB	0.91 m	5.43%	0.50 m	67.39%
**LP-h**	3 m, SIR = 4.0 dB	0.34 m	81.54%	0.11 m	100%
	3 m, SIR = 4.0 dB	0.57 m	34.41%	0.16 m	100%
	1 m, SIR = 1.3 dB	0.60 m	20.71%	0.14 m	100%
**LP-v**	3 m, SIR = 4.0 dB	0.54 m	89.52%	0.23 m	98.10%
	3 m, SIR = 4.0 dB	0.31 m	85.71%	0.21 m	87.62%

**Table 9:** Actual State-of-Art Localization System Comparison (inertial measurement units = IMUs).

Reference	$\begin{array}{c} \mathrm{Number}\mathrm{of}\\ \mathrm{Anchors} \end{array}$	Technology	$\begin{array}{c} \mathrm{Training}\mathrm{Pts}.\\ \mathrm{or}\mathrm{MapSet} \end{array}$	Eq.training pts. for Site Area [19]	Mean Error (m)	Observed Area (m^2^)
[55] Case #1	4	RSSI	21 pts.	16 pts.	1.55	7 × 6.4
[55] Case #2	6	RSSI	18 pts.	19 pts.	1.22	6.3 × 5.1
[55] Case #3	3	RSSI	18 pts.	19 pts.	2.58	6.3 × 5.1
[54] Case “Lab.”	3	CSI	10 + learning	?	0.60	5 × 8
		RSSI	10 + learning	?	0.90	
[54] Case “Corr.”	3	CSI	10 + learning	?	1.00	32.5 × 10
		RSSI	10 + learning	?	1.40	
[13]	4	RSSI + IMUs	30 pts.	7 pts.	1.80	10 × 15
[56]	15	RSSI + IMUs	286 pts.	6 pts.	3.42	70 × 23
[57]	8	RSSI + IMUs	50 pts.	8 pts.	1.00	43.5 × 4.8
[15] Case #1	4	RSSI + IMUs	“Large” Set	?	1.00	19 × 16.3
[15] Case #2	7	RSSI + IMUs	“Large” Set	?	1.00	70 × 23
[58] Case #1	6	RSSI + IMUs	350 pts.	10 pts.	1.00	1250
[58] Case #2	6	RSSI + IMUs	50 pts.	2 pts.	2.75	1250
[19] (mesh)	4	SBA + RSSI	10 + tuning	10 pts.	0.55	2.6 × 3.8
[19] (full)	4	SBA + RSSI	10 + tuning	10 pts.	1.08	5.75 × 6
This work (mesh)	4	SBA + RSSI	**none**	-	0.60	2.6 × 3.8
This work (full)	4	SBA + RSSI	**none**	-	0.87	5.75 × 6

## Abbreviations

The following abbreviations are used in this manuscript:

CDF	Cumulative Distribution Function
COTS	Component-off-the-Shelf
CP	Circular Polarization
CRB	Cramer-Rao Bound
DOA	Direction of Arrival
IMU	Inertial Measurement Units
LP	Linear Polarization
LQI	Link Quality Indicator
ML	Maximum Likelihood estimator
PHY	IEEE Network ISO/OSI Physical Layer
RSSI	Received Signal Strength Indicator
TDoA	Time Direction of Arrival
SBA	Switched-Beam Antenna
SDMA	Space Division Multiplexing Access
